# Burdock Tea Affects Pulmonary Microbiota and Physiology Through Short-Chain Fatty Acids in Wistar Rats

**DOI:** 10.3390/biology14081064

**Published:** 2025-08-16

**Authors:** Xiao-Feng Peng, Jing-Yi Zhu, Li-Zhi Cheng, Wan-Hong Wei, Sheng-Mei Yang, Xin Dai

**Affiliations:** 1 College of Bioscience and Biotechnology, Yangzhou University, 48 East Wenhui Road, Yangzhou 225009, China; mx120231119@stu.yzu.edu.cn (X.-F.P.); mx120231126@stu.yzu.edu.cn (J.-Y.Z.); clz15155938164@outlook.com (L.-Z.C.); whwei@yzu.edu.cn (W.-H.W.); smyang@yzu.edu.cn (S.-M.Y.); 2 Jiangsu Co-Innovation Center for Prevention and Control of Important Animal Infectious Diseases and Zoonoses, Yangzhou University, Yangzhou 225009, China

**Keywords:** burdock tea, SCFAs, gut–lung axis, antioxidant, microbiota, rat

## Abstract

The impact of burdock tea (BT) made from burdock (*Arctium lappa*) roots in normal individuals and animal models remains largely unknown, particularly on lung protection. This study examined responses of oxidative stress, inflammation, and the microbiota within the cecum and the lung to BT treatment in healthy Wistar rats. A middle-dose BT reduced the Chao1 and Shannon indices, and both low and middle doses induced structural alterations in the cecal microbiota. Additionally, low doses increased the abundances of *Phascolarctobacterium*, *Alloprevotella*, *Desulfovibrio*, and the *NK4A214* group. In the lung, middle and high doses increased *Corynebacterium*, with high doses also boosting *Megasphaera* and *Lactobacillus*. Functionally, low doses downregulated the biosynthesis of antibiotics in the cecal microbiota, while middle doses reduced the Epstein–Barr virus and *Escherichia coli* pathogenic infection pathways; additionally, middle and high doses modulated chromosomal proteins and bile acid biosynthesis in the pulmonary microbiota. BT treatment enhanced the content of short-chain fatty acids (SCFAs), upregulated the expression of *GPR43*, and suppressed *NLRP3* expression in both the colon and lung tissues, while concurrently promoting the expression of *ZO-1* and *Occludin*. Furthermore, serum levels of IL-1β and IL-6, as well as tissue levels of MDA, were significantly reduced. Notably, propionate exhibited an inverse correlation with MDA, IL-6, and *NLRP3*, while showing a positive correlation with *ZO-1*. Similarly, acetate was negatively correlated with MDA and *NLRP3* and positively correlated with *ZO-1*. Overall, BT exhibits a nontoxic profile and may protect lung tissue through its antioxidant nature and gut–lung axis mediated by SCFAs.

## Simple Summary

The impact of burdock tea (BT) made from burdock roots on lung protection in normal individuals and animal models is largely unknown. This study examined responses of oxidative stress, inflammation, and the microbiota within the cecum and lung to BT treatment in normal male Wistar rats. The results showed that BT contains numerous bioactive compounds with beneficial functions. It induced alterations in alpha and beta diversities in cecal microbiota, increased the abundances of beneficial microbiota, and altered the enrichment of predicted microbial functions in both the cecum and the lung. It increased the production of short-chain fatty acids (SCFAs) in the cecum, upregulated the expression of SCFA receptors, enhanced barrier function, downregulated the expression of the inflammatory pathway gene *NLRP3*, and reduced inflammation levels in both the colon and lung. It can also downregulate oxidative stress levels. SCFAs were inversely correlated with inflammatory cytokines, oxidative stress levels, and *NLRP3* expression, and positively correlated with *ZO-1* expression. Overall, BT appears to be nontoxic and may protect the lung via the gut–lung axis and its antioxidant nature. The present study provides a theoretical and experimental basis for the further development and application of BT as a daily health supplement.

## 1. Introduction

Burdock (*Arctium lappa*), a biennial Asteraceae species, is a pharmacologically significant plant with dual applications in traditional medicine and global cuisine [1]. While East Asian cultures predominantly utilize its roots, European traditions favor the consumption of tender shoots and leaves. In China, burdock tea (BT) is an herbal beverage made from burdock roots through slicing and drying processes. This beverage is rich in dietary fiber, exhibiting significant anti-inflammatory and antibacterial effects [2]. Additionally, its high polyphenol content demonstrates considerable antioxidant activity [3]. However, because most studies have been conducted in animal models of inflammation or injury, BT’s exact effects on normal individuals and animal models remain unknown. Although there is increasing interest in pulmonary health after the COVID-19 era, the potential protective effects of BT on lung health, as well as the underlying mechanisms by which BT exerts its lung-protective effects, remain unclear. This uncertainty partially restricts the promotion and application of BT as a healthy food.

The gastrointestinal microbiome, the body’s most densely populated microbial community, is crucial for preserving mucosal homeostasis and modulating systemic inflammation through intricate host–microbe interactions [4,5]. When dysbiosis occurs, it can compromise the integrity of the intestinal barrier, allowing microbial translocation that triggers inflammatory responses in far-off organs [6]. Tight junction (TJ) complexes, composed of interacting proteins such as claudins, zonula occludens, and MARVEL-domain proteins, are vital for mucosal defense by controlling paracellular permeability and preventing pathogen infiltration [7,8]. Among these, *Zonula occludens-1* (*ZO-1*) and *Occludin* are key to maintaining the TJ barrier and supporting various epithelial functions, including cell proliferation, repair, and survival [8,9]. Moreover, gut-derived metabolites—especially short-chain fatty acids (SCFAs)—enhance epithelial barrier integrity by reorganizing TJ proteins and activating mitochondrial antioxidant pathways [10,11]. SCFAs exert their effects by binding to G protein-coupled receptors (GPCRs) such as *GPR109A*, *GPR41*, and *GPR43*, which are variably expressed in different cells and tissues [12,13]. In particular, SCFAs elevate colonic *GPR43* levels. Their binding to this receptor induces potassium ion flux, resulting in the hyperpolarization of intestinal epithelial membranes. This process subsequently inhibits *NLRP3* inflammasome activity, diminishes reactive oxygen species, and boosts the expression of tight junction proteins, including *ZO-1* and *Occludin*. This decreases oxidative stress and strengthens the integrity of the intestinal barrier [14].

Clinically, gut microbiota perturbations correlate with heightened susceptibility to pulmonary pathologies, exemplified by early-life depletion of *Bifidobacterium*, *Faecalibacterium*, and *Akkermansia* increasing asthma risk [15]. This gut–lung crosstalk is mediated through microbial metabolites acting as immunometabolic regulators. SCFAs, for instance, modulate dendritic cell maturation and Treg differentiation, thereby ameliorating airway hyperreactivity [16]. Compromised intestinal barriers permit bacterial translocation to pulmonary tissues via pathogen-associated molecular patterns (PAMPs), triggering TNF-α/IL-1β/IL-6-dominated cytokine storms—a mechanistic basis of the gut–lung axis [17].

Building on its established microbiota-modulating properties and the gut–lung axis paradigm, we hypothesize that BT may confer respiratory benefits by modulating the enteric microbiome. In this study, normal male Wistar rats were administered varying doses of BT via gastric gavage over an 8-week period. We systematically evaluated changes in serum inflammatory cytokines, oxidative status, and microbial composition in both the gut and lungs, as well as cecal SCFA content and the expression levels of genes related to TJ proteins and SCFA-associated pathways in intestinal and pulmonary tissues. This study comprehensively investigated the influence of BT on the gut–lung axis, with the objective of elucidating the specific gut microbial mechanisms that mediate pulmonary effects. Our findings provide critical insights that advance the evidence-based development and practical application of BT as a daily functional health care beverage.

## 2. Materials and Methods

### 2.1. Animals and Treatments

Male Wistar rats were procured from Bengbu Yinnojia Biotechnology Co., Ltd. (Bengbu, China), and housed in the experimental animal facility at Yangzhou University under controlled environmental conditions (22 ± 1 °C; 12 h light/dark cycle) with ad libitum access to water and daily feeding of standard rodent chow. Following a 7-day acclimatization period in individual cages, forty male rats were randomly assigned to four groups. The control group received distilled water by gavage, whereas the low-, middle-, and high-dose groups were administered BT suspensions at doses of 3, 6, and 12 g/kg, respectively. Treatments were administered daily at 09:00 for 8 consecutive weeks. Body weights were recorded on a weekly basis. Food intake was evaluated every 7 days by providing 200 g of standard chow daily and measuring the remaining amount to calculate the daily consumption. After a 12 h fast following the final gavage, the rats were anesthetized via tail vein injection. Blood was collected by cardiac puncture and centrifuged (4 °C, 3000× *g* for 30 min) to isolate serum, which was then stored at −70 °C. Visceral organs, including the lungs, liver, kidneys, and spleen, were excised, rinsed with physiological saline, blotted dry, and weighed to determine organ indices (organ weight/final body weight × 100%). Colon, lung, and liver tissues were snap-frozen in sterile cryotubes at −70 °C for subsequent analyses, and cecal contents were aseptically collected and stored at −70 °C for SCFA quantification and 16S rRNA gene sequencing. All procedures were performed in accordance with the guidelines approved by the Animal Care and Use Committee of the Faculty of Veterinary Medicine at Yangzhou University (No. NSFC2020-SKXY-6).

### 2.2. Preparation and Component Detection of BT

#### 2.2.1. Preparation of BT

BT was prepared with modifications to the method described by Feng et al. [18]. Briefly, 18 g of dry burdock root was soaked in 180 mL of ultrapure water at 80–90 °C for 30 min and extracted twice by a 100-mesh sieve under atmospheric pressure. The combined extracts were concentrated to 15 mL using a rotary evaporator (DSB-2100, Tokyo Rikakikai Co., Ltd., Tokyo, Japan) at a reduced pressure of −0.08 MPa and a temperature of 68 ± 1 °C, yielding a stock solution with a mass-to-volume equivalence of 1.2 g of raw material per mL. Serial dilutions with distilled water were performed to prepare gavage solutions at concentrations of 0.3 g/mL for the low-dose group, 0.6 g/mL for the middle-dose group, and 1.2 g/mL for the high-dose group. These solutions were administered via oral gavage at a dosage of 1 mL per 100 g of body weight, while the control group received an equivalent volume of distilled water. The dosages employed in the present study were based on those reported by Feng [19], with certain modifications.

#### 2.2.2. Component Detection of BT

A measured portion of BT extract was added to a 2 mL tube with 600 μL of methanol containing 4 μg/mL 2-chloro-L-phenylalanine and vortexed for 30 s. The sample was homogenized with steel beads (55 Hz, 60 s), ultrasonicated at room temperature for 15 min, and then centrifuged at 12,000 rpm and 4 °C for 10 min. The supernatant was filtered through a 0.22 μm membrane and transferred to a vial for LC-MS. Chromatography was carried out on a Vanquish UHPLC system (Thermo Fisher Scientific, Waltham, MA, USA) with an ACQUITY UPLC HSS T3 column (2.1 × 100 mm, 1.8 μm; Waters, Milford, MA, USA) at 40 °C. For the positive-ion mode, mobile phases were 0.1% formic acid in water (A2) and acetonitrile (B2) with a gradient: 8% B2 (0–1 min), 8% to 98% B2 (1–8 min), 98% B2 (8–10 min), 98% to 8% B2 (10–10.1 min), and 8% B2 (10.1–12 min). For the negative-ion mode, acetonitrile (B3) and 5 mM ammonium formate in water (A3) followed the same process. The flow rate was 0.3 mL/min with a 2 μL injection volume. Mass spectrometric detection was performed on Thermo Q Exactive Focus (Thermo Fisher Scientific, USA) with electrospray ionization in polarity-switching mode. Key parameter settings included spray voltages of +3.50 kV and −2.50 kV, sheath gas at 40 units, auxiliary gas at 10 units, a capillary temperature of 325 °C, and full MS scans at 70,000 FWHM (*m*/*z* 100–1000). Data-dependent MS/MS selected the top three precursor ions for HCD fragmentation at 30 eV and 17,500 FWHM with dynamic exclusion to avoid redundant spectra.

### 2.3. Determination of Immunoglobulin

Serum concentrations of interleukin-1 beta (IL-1β) and interleukin-6 (IL-6) were quantified using commercially available enzyme-linked immunosorbent assay (ELISA) kits from Jianglai Bioengineering Institute (Shanghai, China) according to the manufacturer’s protocols and established methodologies. The intra- and inter-assay coefficients of variation for both biomarkers were maintained below 10%.

### 2.4. Detection of Oxidative Stress Levels

Lung, liver, and colon tissue samples were homogenized in ice-cold phosphate-buffered saline and subsequently centrifuged at 3000× *g* for 10 min at 4 °C. The resulting supernatants were collected for biochemical analyses. Superoxide dismutase (SOD) activity, catalase (CAT) activity, and malondialdehyde (MDA) concentration were quantified using commercially available assay kits (Yfxbio Biotech, Nanjing, China) according to the manufacturer’s protocols. Absorbance measurements were performed in triplicate using a microplate reader with the following wavelength settings: 450 nm for SOD, 240 nm for CAT, and 532 nm for MDA.

### 2.5. Quantitative Real-Time PCR Analysis

Total RNA was extracted from colon and lung samples using a previously established protocol [20]. An aliquot of 1 μg was subsequently reverse-transcribed into cDNA using the PrimeScript™ 1st Strand cDNA Synthesis Kit (R323, Vazyme Biotech, Nanjing, China) in accordance with the manufacturer’s guidelines. Quantitative PCR amplification was carried out on a Real-Time PCR System (Applied Biosystems, Foster City, CA, USA) employing SYBR Premix EX Taq™ II (TaKaRa, Dalian, China). Gene-specific primers for *ZO-1*, *Occludin*, *GPR43*, and *NLRP3* were designed via NCBI Primer-BLAST by targeting the respective rat coding DNA sequences (Table S1). Thermal cycling conditions were as follows: an initial denaturation at 95 °C for 30 s, followed by 40 cycles of 95 °C for 10 s and 60 °C for 30 s. Gene expression levels were normalized to β-actin [21] and quantified using the 2^−ΔΔCt^ method [22], with no amplification observed in either no-template or non-reverse-transcription controls. 

### 2.6. SCFA Content Measurement

SCFA concentrations—including acetate, propionate, butyrate, isobutyrate, valerate, and isovalerate—in cecal contents were quantified using gas chromatography–mass spectrometry (GC-MS). The methodology was based on the protocol described by Shen et al. [23]. Samples and standards were analyzed on an Agilent 7080A GC system (Agilent, Santa Clara, CA, USA) equipped with a flame ionization detector (FID) and a Supelco Nukol fused silica capillary column (30 m × 0.25 mm, with a film thickness of 0.25 μm). Peak areas were quantified using Agilent ChemStation (Agilent Technologies, Waldbronn, Germany), and SCFA concentrations were determined by comparing sample peak areas against standard calibration curves.

### 2.7. 16S rRNA Gene Sequencing and Bioinformatic Analysis of Cecal and Pulmonary Microbiota

To minimize sequencing costs, cecal content and lung tissue samples from 6 rats randomly selected were subjected to 16S rRNA high-throughput sequencing in a company (Novogene Co., Ltd., Beijing, China). Sequencing was performed on an Illumina NovaSeq 6000 platform (Illumina, San Diego, CA, USA), generating raw sequencing data that underwent quality control processing: raw reads were spliced and filtered to produce clean data, which were then clustered into amplicon sequence variants (ASVs) using DADA2 in the QIIME2 (2022.02) software. Taxonomic annotation of ASVs was performed against the SILVA reference database (v138) to determine species composition and abundance profiles. The sequence data are available at the NIH Sequence Read Archive with the Bioproject ID of PRJNA1261695 (https://submit.ncbi.nlm.nih.gov/subs/bioproject/ (accessed on 12 May 2025)). Microbial diversity analyses included calculation of α-diversity indices (Chao1, Good’s coverage, observed features, Shannon, Simpson, and Pielou) at the ASV level by QIIME2. Linear discriminant analysis effect size (LEfSe) with an LDA score threshold ≥3 identified statistically significant biomarkers across the four groups using the Huttenhower Lab server platform (http://huttenhower.sph.harvard.edu/galaxy/ (accessed on 23 February 2024)). Biological functional prediction of ASV was predicted and annotated using the Kyoto Encyclopedia of Genes and Genomes (KEGG) pathway database by the Tax4Fun program.

### 2.8. Statistical Analysis

Body weight changes in Wistar rats were analyzed using repeated-measures ANOVA. Daily food intake, organ indices, cecal SCFAs, gene expression, and markers of antioxidant and inflammation status were assessed via one-way ANOVA with Tukey’s post hoc test after verification of normality and homogeneity of variance. Intergroup differences in α-diversity indices, amplicon sequence variant (ASV) biomarker abundances, and predicted microbiota pathway enrichment profiles were evaluated using nonparametric Kruskal–Wallis tests followed by pairwise comparisons implemented with the tidyverse R package (v4.4.3). The Benjamini–Hochberg false discovery rate (FDR) was controlled at 0.1. β-diversity analysis of the cecal microbiota was performed with permutational multivariate ANOVA (PERMANOVA) based on Bray–Curtis dissimilarity matrices computed via the nested adonis function in the vegan R package (v4.4.3). Principal coordinate analysis (PCoA), implemented using the ape package, was applied to visualize microbial community structures at the ASV level, with analysis of similarities (ANOSIM) (using 999 permutations) quantifying group separations. PCoA and ANOSIM visualizations were generated using the ggplot2 R package (v4.4.3). Spearman correlation analysis, with FDR adjustment, was performed using the psych R package (v4.4.3), and the results were plotted via the corrplot R package (v4.4.3). Statistical significance was defined as a *p*-value of less than 0.05 or an FDR-adjusted *p*-value of less than 0.1. All ANOVA analyses were conducted using IBM SPSS Statistics 26 (IBM, Armonk, NY, USA).

## 3. Results

### 3.1. The Components of BT

**Figure 1:**
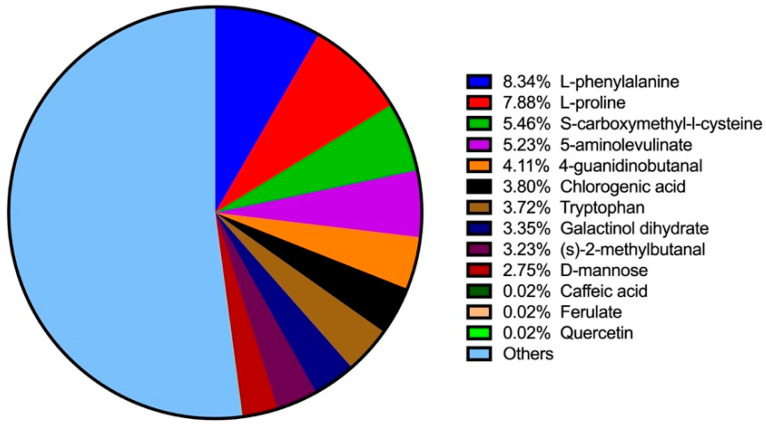
Top 10 ingredients and additional phenolic compounds in BT detected by LC–MS.

Using LC–MS analysis, 649 components were identified in BT samples (see Figure S1 for substance peak diagrams in both the positive- and negative-ion modes). These components primarily consisted of amino acids, alkaloids, flavonoids, lipids, carbohydrates, phenylpropanoids, phenolics, vitamins, steroids, terpenoids, organic acids, and short peptides. The ten most prevalent constituents were L-phenylalanine (8.34%), L-proline (7.88%), S-carboxymethyl-L-cysteine (5.46%), 5-aminolevulinate (5.23%), 4-guanidinobutanal (4.11%), chlorogenic acid (3.80%), tryptophan (3.72%), galactinol dihydrate (3.35%), (S)-2-methylbutanal (3.23%), and D-mannose (2.75%) (Figure 1). Additional bioactive phenolic compounds identified included caffeic acid (0.023%), ferulate (0.02%), and quercetin (0.02%).

### 3.2. Effect of BT on Growth and Organ Indices

There were no significant differences in body weight (*F*_3,32_ = 1.456, *p* = 0.245) or daily food consumption (*F*_3,36_ = 0.267, *p* = 0.849) among the four groups (Table S2 and Figure S2). Similarly, the liver, kidney, lung, and spleen indices did not differ significantly (*F*_3,36_ = 2.411, *p* = 0.083; *F*_3,36_ = 2.056, *p* = 0.123; *F*_3,36_ = 1.847, *p* = 0.156; and *F*_3,36_ = 0.755, *p* = 0.527, respectively; Table S2).

### 3.3. Effect of BT on Immunoglobulin Levels

**Figure 2:**
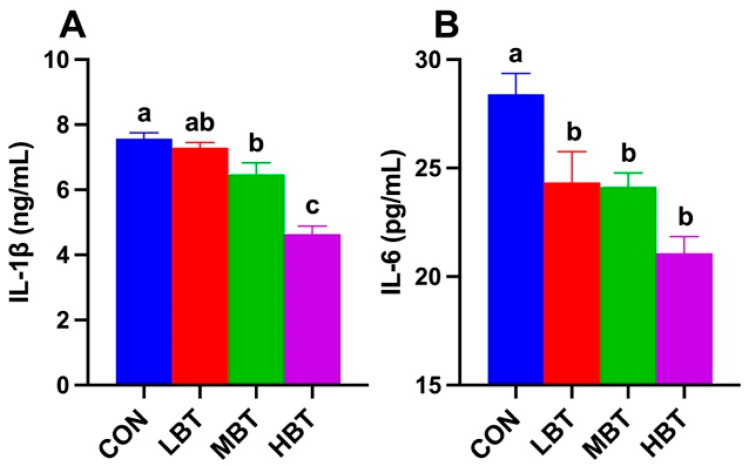
Effects of BT on serum IL-1β (**A**) and IL-6 (**B**) levels in Wistar rats (*n* = 10). CON: control; LBT: low-dose BT; MBT: middle-dose BT; HBT: high-dose BT. Error bars indicate standard errors. Groups marked with different letters differ significantly (*p* < 0.05).

Serum IL-1β levels in both middle- and high-dose BT groups were significantly lower than those in the control group (*F*_3,36_ = 29.068, *p* < 0.001; *p* = 0.042 and *p* < 0.001, respectively; Figure 2A). Serum IL-6 levels in low-, middle-, and high-dose BT groups were all significantly lower than those in the control group (*F*_3,36_ = 9.214, *p* < 0.001; *p* = 0.030 and 0.021 and *p* < 0.001, respectively; Figure 2B).

### 3.4. Effect of BT on Levels of MDA, SOD, and CAT Activity

**Figure 3:**
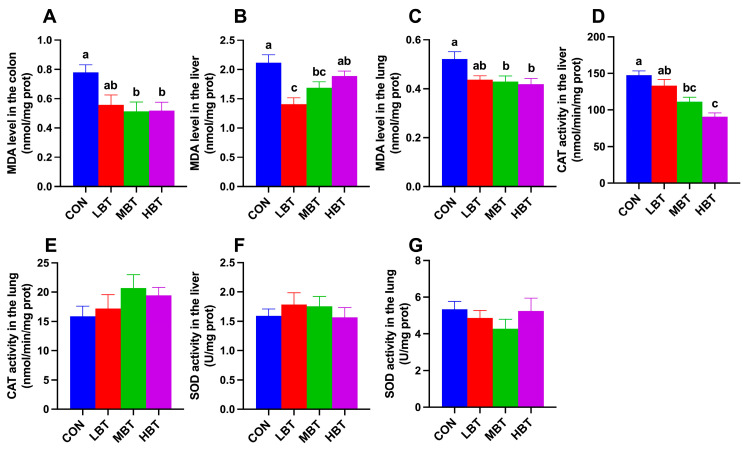
Effect of BT on the MDA (**A**) level in the colon, and MDA (**B**,**C**), CAT (**D**,**E**), and SOD (**F**,**G**) activity in the liver and lung of Wistar rats (*n* = 10). CON: control; LBT: low-dose BT; MBT: middle-dose BT; HBT: high-dose BT. Error bars indicate standard errors. Groups marked with different letters differ significantly (*p* < 0.05).

In the colon, MDA levels in the middle- and high-dose BT groups were significantly lower than those in the control group (*F*_3,36_ = 4.412, *p* = 0.010; *p* = 0.018 and 0.020, respectively; Figure 3A). In the liver, both the low- and middle-dose BT groups exhibited significantly lower MDA levels compared to the control group (*F*_3,36_ = 5.049, *p* = 0.005; *p* < 0.001 and *p* = 0.045, respectively; Figure 3B). As observed in the colon, pulmonary MDA levels in the middle- and high-dose BT groups were reduced relative to the control group (*F*_3,36_ = 3.279, *p* = 0.032; *p* = 0.042 and 0.019, respectively; Figure 3C). Additionally, liver CAT activity in the middle- and high-dose BT groups was lower than that in the control group (*F*_3,36_ = 14.193, *p* < 0.001; *p* = 0.002 and *p* < 0.001, respectively; Figure 3D). No significant differences were observed in liver SOD activity or lung SOD and CAT activity across the four groups (*F*_3,36_ = 0.446, *p* = 0.722; *F*_3,36_ = 0.830, *p* = 0.486; and *F*_3,36_ = 1.188, *p* = 0.328, respectively; Figure 3E–G).

### 3.5. Effect of BT on Cecal Microbiota

#### 3.5.1. ASV Analysis of Cecal Microbiota

**Figure 4:**
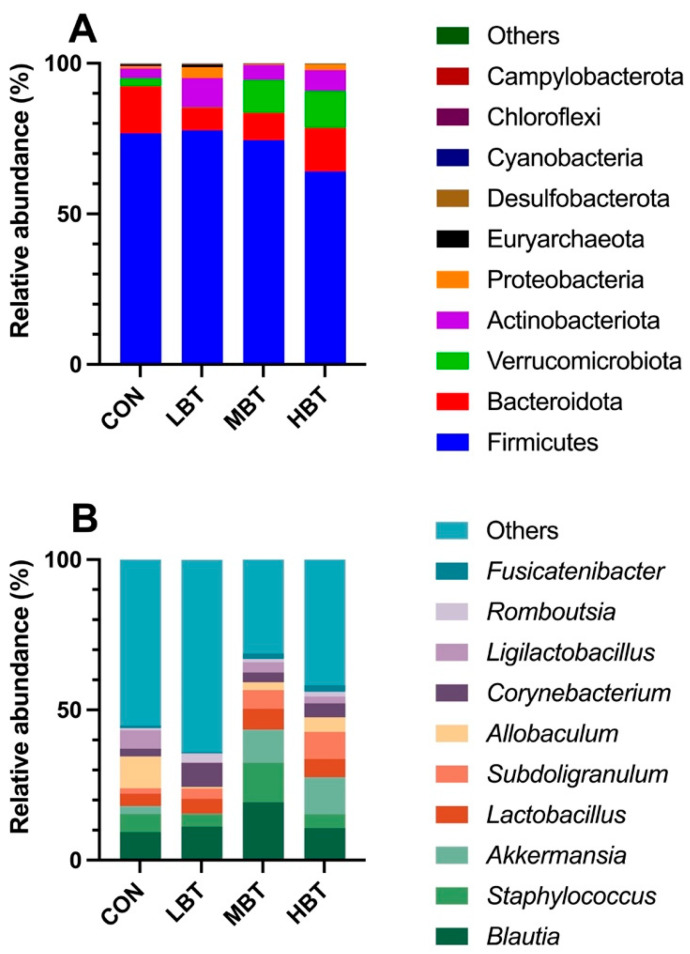
The top 10 taxonomic units at the phylum (**A**) and genus (**B**) levels in the cecum microbial community of Wistar rats (*n* = 6). CON: control; LBT: low-dose BT; MBT: middle-dose BT; HBT: high-dose BT.

A total of 17 phyla, 23 classes, 58 orders, 113 families, 241 genera, and 1992 ASVs were identified in the cecum samples of the four experimental groups, with Good’s coverage reaching 1. The dominant phyla were Firmicutes (73.32 ± 2.97%), followed by Bacteroidota (11.63 ± 2.04%), Verrucomicrobiota (6.56 ± 2.37%), Actinobacteriota (6.16 ± 0.93%), and Proteobacteria (1.64 ± 0.61%) (Figure 4A). At the genus level, *Blautia* was predominant (12.66 ± 1.54%), followed by *Staphylococcus* (6.96 ± 1.51%), *Akkermansia* (6.56 ± 2.37%), *Lactobacillus* (5.49 ± 1.09%), and *Subdoligranulum* (5.12 ± 1.27%) (Figure 4B).

#### 3.5.2. Differences in Diversities of Cecal Microbial Community

In the middle-dose BT group, the observed features (χ^2^ = 10.651, effect size = 0.383, *p* = 0.014; FDR-adjusted *p* = 0.060) and Chao1 index (χ^2^ = 10.420, effect size = 0.371, *p* = 0.015; FDR-adjusted *p* = 0.061) were lower than those in the control group (Table 1). In contrast, no significant differences were observed among the four groups regarding the Simpson index (χ^2^ = 5.563, *p* = 0.135) or the Pielou index (χ^2^ = 7.309, *p* = 0.063) (Table 1).

**Figure 5:**
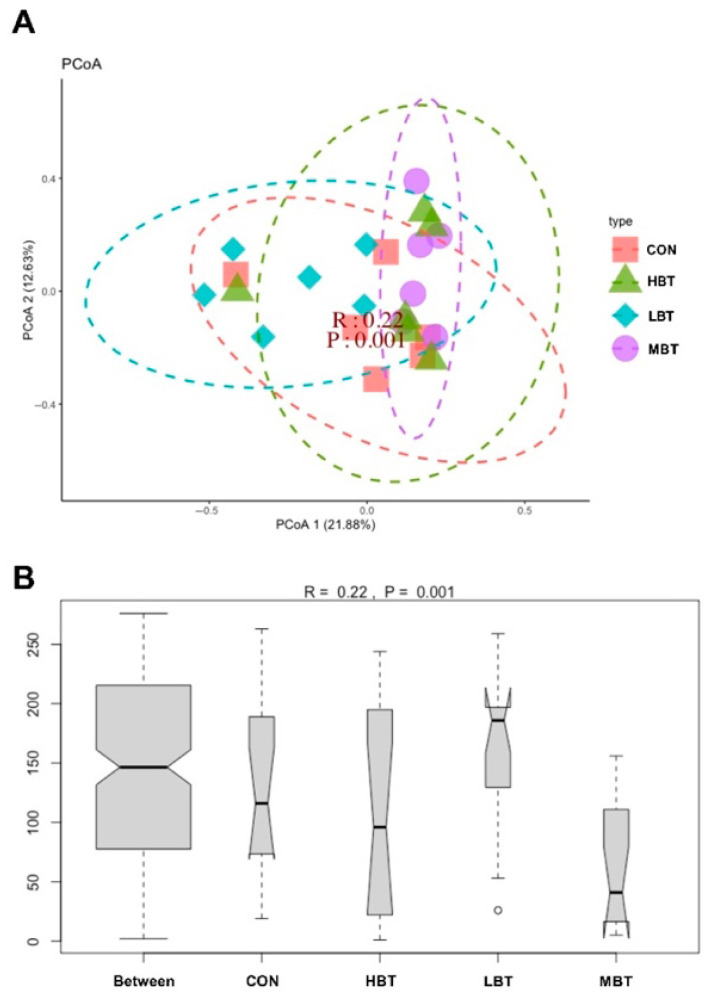
Effect of BT on the cecal microbial community structure of Wistar rats (*n* = 6). (**A**) Principal coordinate analysis (PCoA) plot; (**B**) ANOSIM analysis plot. CON: control; LBT: low-dose BT; MBT: middle-dose BT; HBT: high-dose BT.

PERMANOVA analysis showed that eight weeks of BT administration significantly altered the beta diversity of the cecal microbial community (*F* = 1.8992, *p* = 0.001), with the low- and middle-dose BT groups differing significantly from the control group (*p* = 0.021 and *p* = 0.028, respectively). Furthermore, principal coordinate analysis (PCoA) based on Bray–Curtis dissimilarity revealed that the first and second principal axes (PCoA1 and PCoA2) accounted for 21.88% and 12.63% of the variation in the cecal microbiome structure after eight weeks of BT administration (Figure 5A). Although the ordination plot did not completely segregate the control, low-, middle-, and high-dose BT groups, ANOSIM demonstrated that the intergroup differences exceeded the intragroup differences observed in the control, middle-, and high-dose BT cohorts (*R* = 0.22, *p* = 0.001) (Figure 5B).

#### 3.5.3. Differences in Abundances of Cecal Microbiota

**Figure 6:**
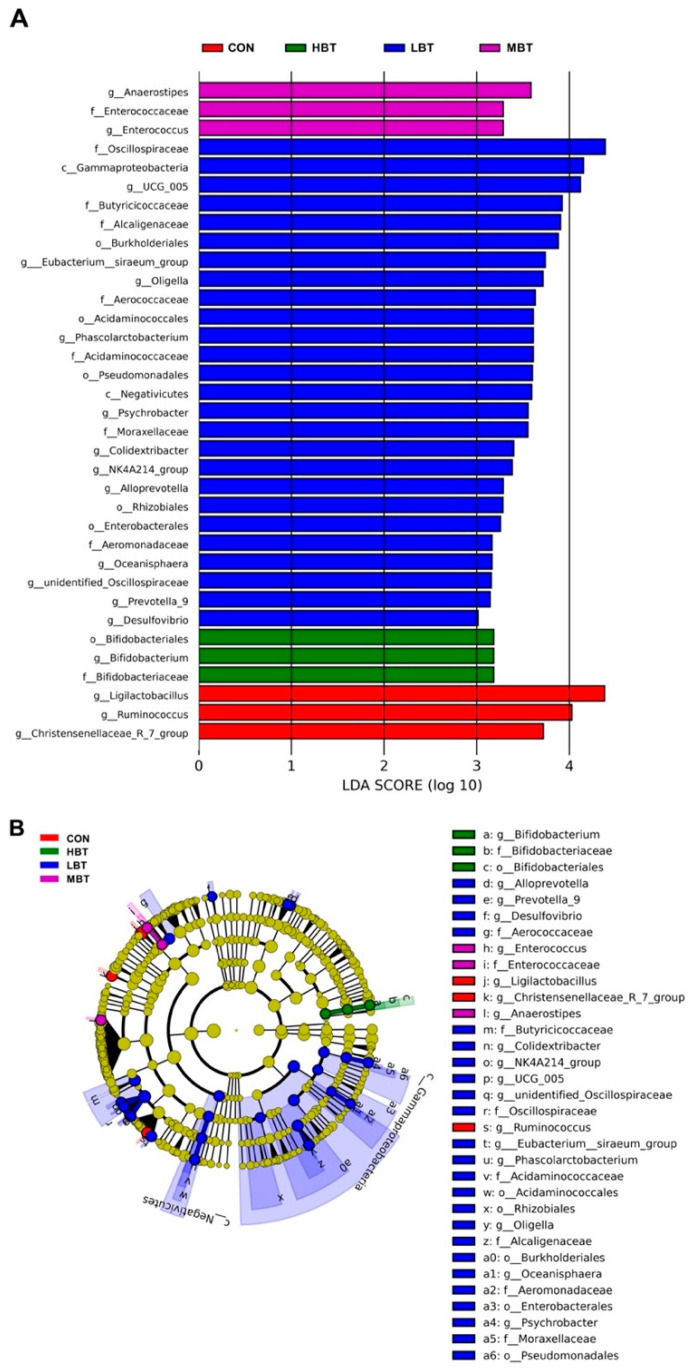
Taxa identified as biomarkers in the cecal microbial community of Wistar rats (*n* = 6). (**A**) Bar plot displaying taxa with significant LDA scores (LDA > 3); (**B**) cladogram of phylogenetically enriched taxa across four groups. CON: control; LBT: low-dose BT; MBT: middle-dose BT; HBT: high-dose BT.

**Figure 7:**
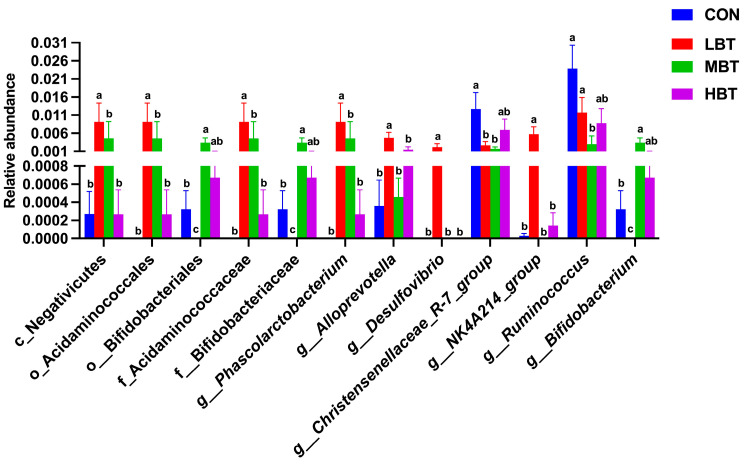
Differences in the cecal microbial abundances at the class, order, family, and genus levels in response to BT treatment in Wistar rats (*n* = 6). CON: control; LBT: low-dose BT; MBT: middle-dose BT; HBT: high-dose BT. Error bars indicate standard errors. Groups marked with different letters differ significantly (FDR-adjusted *p* < 0.1).

In the low-dose BT group, the relative abundances of the class Negativicutes (χ^2^ = 10.702, effect size = 0.385, *p* = 0.013; FDR-adjusted *p* = 0.037), the order Acidaminococcales (χ^2^ = 14.470, effect size = 0.574, *p* = 0.002; FDR-adjusted *p* = 0.017), and the family Acidaminococcaceae (χ^2^ = 14.470, effect size = 0.574, *p* = 0.002; FDR-adjusted *p* = 0.017) were significantly higher than those in the control group (Figure 6A,B and Figure 7). In the low- and middle-dose BT groups, the relative abundances of the order Bifidobacteriales and the family Bifidobacteriaceae were significantly higher than those in the control group (χ^2^ = 13.061, effect size = 0.503, *p* = 0.005; FDR-adjusted *p* = 0.057 and 0.067, respectively) (Figure 6A,B and Figure 7). Additionally, the relative abundances of the orders Rhizobiales, Enterobacterales, Burkholderiales, and Pseudomonatales, along with the families Moraxellaceae, Aerococcaceae, Oscillospiraceae, Alcaligenaceae, Enterococcaceae, and Butyricicoccaceae, differed significantly from the control group following BT treatment (FDR-adjusted *p* < 0.1) (Table S3).

In the low-dose BT group, the relative abundances of the genera *Phascolarctobacterium* (χ^2^ = 14.470, effect size = 0.574, *p* = 0.002; FDR-adjusted *p* = 0.017), *Alloprevotella* (χ^2^ = 8.708, effect size = 0.285, *p* = 0.033; FDR-adjusted *p* = 0.077), *Desulfovibrio* (χ^2^ = 13.658, effect size = 0.533, *p* = 0.003; FDR-adjusted *p* = 0.028), and *NK4A214 group* (χ^2^ = 13.649, effect size = 0.532, *p* = 0.003; FDR-adjusted *p* = 0.033) were higher than those in the control group. In the low- and middle-dose BT groups, the relative abundance of the genus *Bifidobacterium* was higher than that in the control group (χ^2^ = 13.061, effect size = 0.503, *p* = 0.005; FDR-adjusted *p* = 0.057 and 0.067, respectively). Conversely, the relative abundance of the genus *Ruminococcus* (χ^2^ = 10.096, effect size = 0.355, *p* = 0.018; FDR-adjusted *p* = 0.078) in the middle-dose BT groups and that of the *Christensenellaceae-R7 group* (χ^2^ = 7.867, effect size = 0.243, *p* = 0.049; FDR-adjusted *p* = 0.092 and 0.030, respectively) in the low- and middle-dose BT groups were lower than those in the control group (Figure 6A,B and Figure 7). Additionally, the abundances of the genera *Prevotella* 9, *Colidextribacter*, *[Eubacterium] siraeum group*, *unidentified Oscillospiraceae*, *Enterococcus*, *Anaerostipes*, and *UCG-005* differed significantly from the control group following BT treatment (FDR-adjusted *p* < 0.1; Table S3).

#### 3.5.4. Differences in Abundances of Cecal Predicted Microbial Function

**Figure 8:**
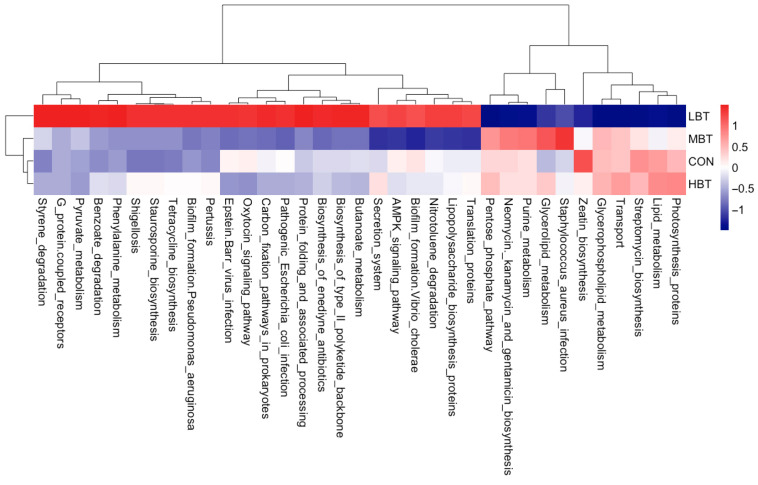
KEGG pathways with significantly varied enrichment in the cecal microbiota in response to the administration of BT in Wistar rats (*n* = 6). CON: control; LBT: low-dose BT; MBT: middle-dose BT; HBT: high-dose BT.

The abundances of 35 KEGG level-3 pathways within the cecal bacterial communities were altered following eight weeks of BT administration (Figure 8). Specifically, the benzoate degradation (χ^2^ = 9.347, effect size = 0.317, *p* = 0.025; FDR-adjusted *p* = 0.039), butanoate metabolism (χ^2^ = 10.307, effect size = 0.365, *p* = 0.016; FDR-adjusted *p* = 0.091), phenylalanine metabolism (χ^2^ = 8.447, effect size = 0.272, *p* = 0.038; FDR-adjusted *p* = 0.092), and pyruvate metabolism (χ^2^ = 8.620, effect size = 0.281, *p* = 0.035; FDR-adjusted *p* = 0.091) pathways were more dominant in the low-dose BT group compared to the control group. Glycerolipid metabolism was more abundant in the middle-dose group than in the control group (χ^2^ = 9.947, effect size = 0.347, *p* = 0.019; FDR-adjusted *p* = 0.092). In contrast, the neomycin, kanamycin, and gentamicin biosynthesis (χ^2^ = 10.847, effect size = 0.392, *p* = 0.013; FDR-adjusted *p* = 0.061) and streptomycin biosynthesis (χ^2^ = 7.847, effect size = 0.242, *p* = 0.049; FDR-adjusted *p* = 0.078) pathways were less dominant in the low-dose BT group relative to the control group. Moreover, the Epstein–Barr virus infection pathway was less abundant in the middle- and high-dose BT groups than in the control group (χ^2^ = 12.671, effect size = 0.484, *p* = 0.005; FDR-adjusted *p* = 0.030 and 0.068, respectively). The pathogenic *Escherichia coli* infection pathway (χ^2^ = 12.120, effect size = 0.456, *p* = 0.007; FDR-adjusted *p* = 0.025) was less abundant in the middle-dose BT group relative to the control group (Table S4). Furthermore, the abundances of pathways related to G protein-coupled receptors, pertussis, protein folding and associated processing, styrene degradation, lipid metabolism, photosynthesis proteins, transport, the oxytocin signaling pathway, the pentose phosphate pathway, the biosynthesis of enediyne antibiotics, and the biosynthesis of the type II polyketide backbone exhibited significant differences compared to the control group following BT treatment (FDR-adjusted *p* < 0.1) (Table S4).

#### 3.5.5. Relationship Between Abundances of Cecal Microbiota and Predicted Microbial Function

**Figure 9:**
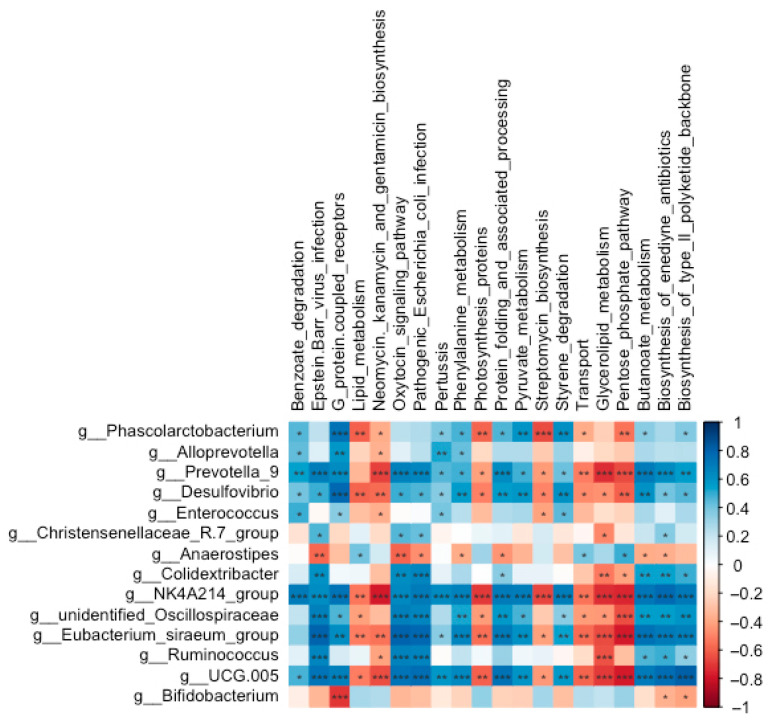
Correlations between the abundances of significantly altered genera and the enrichment of predicted functional pathways in the cecal microbial community of Wistar rats (*n* = 6). * means FDR-adjusted *p* < 0.1, ** means FDR-adjusted *p* < 0.01, and *** means FDR-adjusted *p* < 0.001.

The neomycin, kanamycin, and gentamicin biosynthesis pathway was negatively correlated with the abundances of *Desulfovibrio* (*r* = −0.56, FDR-adjusted *p* = 0.004) and the *NK4A214 group* (*r* = −0.78, FDR-adjusted *p* < 0.001) (Figure 9). Streptomycin biosynthesis displayed significant negative correlations with *Phascolarctobacterium* (*r* = −0.65, FDR-adjusted *p* = 0.001), *Desulfovibrio* (*r* = −0.49, FDR-adjusted *p* = 0.014), and the *NK4A214 group* (*r* = −0.67, FDR-adjusted *p* < 0.001). In contrast, benzoate degradation exhibited positive correlations with *Phascolarctobacterium* (*r* = 0.46, FDR-adjusted *p* = 0.025), *Alloprevotella* (*r* = 0.43, FDR-adjusted *p* = 0.034), *Desulfovibrio* (*r* = 0.41, FDR-adjusted *p* = 0.048), and the *NK4A214 group* (*r* = 0.71, FDR-adjusted *p* < 0.001). Additionally, both the pathogenic *Escherichia coli* infection and Epstein–Barr virus infection pathways were positively correlated with *Ruminococcus* (*r* = 0.67, FDR-adjusted *p* < 0.001; *r* = 0.65, FDR-adjusted *p* = 0.001, respectively) and the *Christensenellaceae-R7 group* (*r* = 0.43, FDR-adjusted *p* = 0.037; *r* = 0.46, FDR-adjusted *p* = 0.023, respectively).

### 3.6. Effect of BT on Pulmonary Microbiota

#### 3.6.1. ASV Analysis of Pulmonary Microbiota

**Figure 10:**
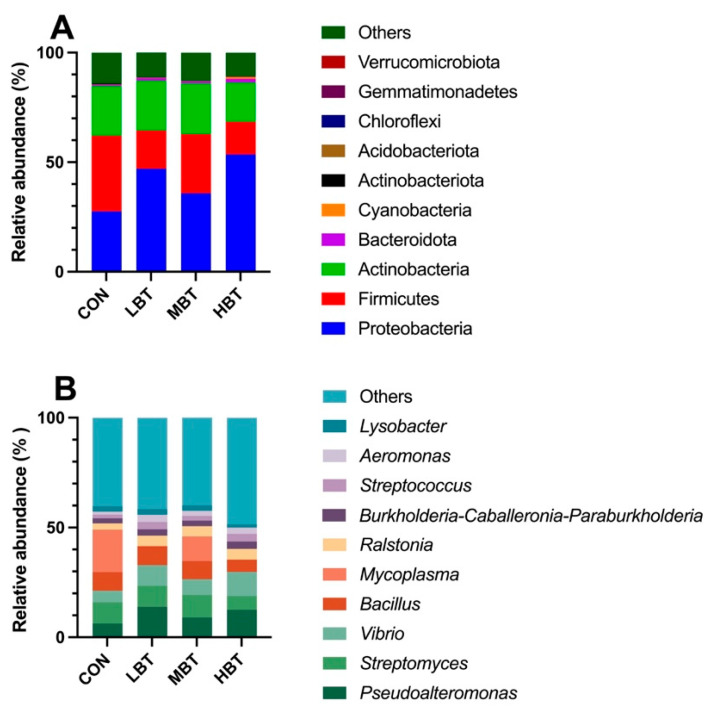
The top 10 taxa at the phylum (**A**) and genus (**B**) levels in the pulmonary microbial community of Wistar rats (*n* = 6). CON: control; LBT: low-dose BT; MBT: middle-dose BT; HBT: high-dose BT.

In total, 44 phyla, 94 classes, 190 orders, 294 families, 500 genera, and 4669 ASVs were identified in the lung samples across the four experimental groups. Good’s coverage index reached 1, suggesting complete sampling coverage. At the phylum level, Proteobacteria (41.01 ± 4.89%) were the most predominant, followed by Firmicutes (23.46 ± 4.08%), Actinobacteria (21.60 ± 4.14%), and Bacteroidota (1.07 ± 0.20%) (Figure 10A). At the genus level, the dominant taxa were *Pseudoalteromonas* (10.45 ± 2.09%), followed by *Streptomyces* (8.93 ± 2.00%), *Vibrio* (8.20 ± 1.52%), *Bacillus* (7.73 ± 1.72%), and *Mycoplasma* (7.67 ± 4.64%) (Figure 10B).

#### 3.6.2. Differences in Diversities of Pulmonary Microbial Community

No significant differences were observed in the observed features (χ^2^ = 0.647, *p* = 0.886) or the Chao1 (χ^2^ = 0.647, *p* = 0.886), Shannon (χ^2^ = 0.780, *p* = 0.854), Simpson (χ^2^ = 0.741, *p* = 0.864) and Pielou indices (χ^2^ = 0.087, *p* = 0.993) among the four groups (Table S5). The PERMANOVA analysis showed that BT administration for eight weeks did not significantly alter the beta diversity of the lung microbial community (*F* = 0.6418, *p* = 0.758).

#### 3.6.3. Differences in Abundances of Pulmonary Microbiota

**Figure 11:**
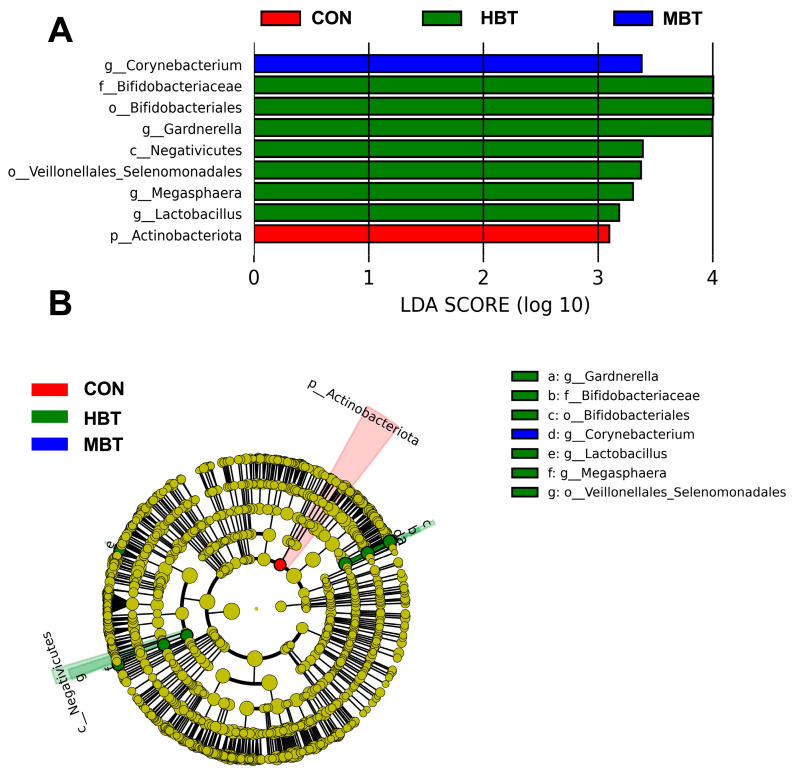
Taxa identified as biomarkers in the pulmonary microbial community of Wistar rats (*n* = 6). (**A**) Bar plot displaying taxa with significant LDA scores (LDA > 3); (**B**) cladogram of phylogenetically enriched taxa across three groups. CON: control; MBT: middle-dose BT; HBT: high-dose BT.

**Figure 12:**
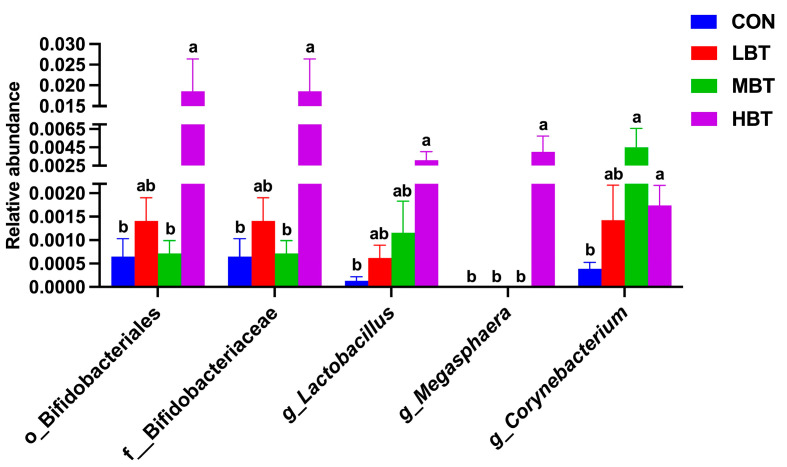
Differences in the pulmonary microbial abundances at the order, family, and genus levels in Wistar rats (*n* = 6). CON: control; LBT: low-dose BT; MBT: middle-dose BT; HBT: high-dose BT. Error bars indicate standard errors. Groups marked with different letters differ significantly (FDR-adjusted *p* < 0.1).

The relative abundances of the order Bifidobacteriales (χ^2^ = 8.644, effect size = 0.282, *p* = 0.034; FDR-adjusted *p* = 0.091), the family Bifidobacteriaceae (χ^2^ = 8.644, effect size = 0.282, *p* = 0.034; FDR-adjusted *p* = 0.091), and the genera *Lactobacillus* (χ^2^ = 8.672, effect size = 0.284, *p* = 0.034; FDR-adjusted *p* = 0.069) and *Megasphaera* (χ^2^ = 13.658, effect size = 0.533, *p* = 0.003; FDR-adjusted *p* = 0.028) in the high-dose BT group were significantly higher than those in control group (Figure 11A,B and Figure 12). Moreover, the relative abundances of the genus *Corynebacterium* (χ^2^ = 8.595, effect size = 0.280, *p* = 0.035; FDR-adjusted *p* = 0.078 and 0.092, respectively) in both the middle- and high-dose BT groups were elevated compared to the control group (Figure 11A,B and Figure 12). In addition, a significant difference was detected in the order Veillonellales–Selenomonadales between the BT-treated and control groups (FDR-adjusted *p* < 0.1; Table S3).

#### 3.6.4. Differences in Abundances of Pulmonary Microbial Predicted Functions

**Figure 13:**
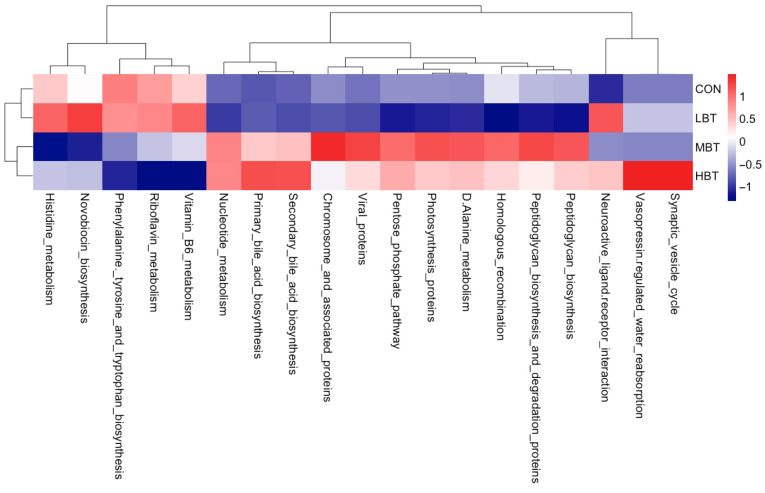
KEGG pathways with the significantly altered enrichment in the pulmonary microbial community in response to the administration of BT in Wistar rats (*n* = 6). CON: control; LBT: low-dose BT; MBT: middle-dose BT; HBT: high-dose BT.

The abundances of 19 KEGG level-3 pathways in the lung bacterial communities were altered following the administration of BT for eight weeks (Figure 13). Specifically, the chromosome and associated proteins (χ^2^ = 9.740, effect size = 0.337, *p* = 0.021; FDR-adjusted *p* = 0.061) and the D-alanine metabolism (χ^2^ = 11.420, effect size = 0.421, *p* = 0.010; FDR-adjusted *p* = 0.039) pathways were more dominant in the middle-dose BT group compared to the control group. The primary bile acid biosynthesis pathway (χ^2^ = 9.980, effect size = 0.349, *p* = 0.019; FDR-adjusted *p* = 0.061) was more prevalent in the high-dose BT group than in the control group. Conversely, compared to controls, the middle- and high-dose BT groups demonstrated a lower abundance of riboflavin metabolism (χ^2^ = 14.447, effect size = 0.572, *p* = 0.002; FDR-adjusted *p* = 0.098 and 0.025, respectively), while the high-dose BT group exhibited reduced levels of vitamin B6 metabolism (χ^2^ = 8.740, effect size = 0.287, *p* = 0.033; FDR-adjusted *p* = 0.061) (Table S6). Additionally, the pathways involved in neuroactive ligand–receptor interaction, viral proteins, vasopressin-regulated water reabsorption, and synaptic vesicle cycle pathways differed significantly from the control group following BT treatment (FDR-adjusted *p* < 0.1 for all; Table S6).

#### 3.6.5. Relationship Between Abundances of Pulmonary Microbiota and Predicted Microbial Functions

**Figure 14:**
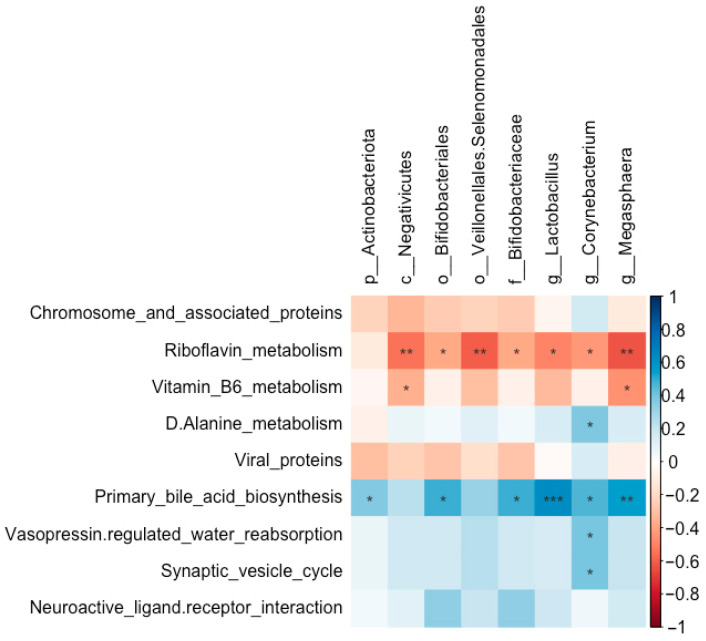
Correlations between the abundances of significantly altered taxa and the enrichment of predicted functional pathways in the pulmonary microbial community of Wistar rats (*n* = 6). * means FDR-adjusted *p* < 0.1, ** means FDR-adjusted *p* < 0.01, and *** means FDR-adjusted *p* < 0.001.

The abundances of Bifidobacteriales (*r* = 0.49, FDR-adjusted *p* = 0.015), Bifidobacteriaceae (*r* = 0.49, FDR-adjusted *p* = 0.015), *Lactobacillus* (*r* = 0.63, FDR-adjusted *p* = 0.001), *Corynebacterium* (*r* = 0.46, FDR-adjusted *p* = 0.022), and *Megasphaera* (*r* = 0.55, FDR-adjusted *p* = 0.005) were all positively correlated with primary bile acid biosynthesis (Figure 14). In contrast, the abundances of Bifidobacteriales (*r* = −0.38, FDR-adjusted *p* = 0.064), Bifidobacteriaceae (*r* = −0.38, FDR-adjusted *p* = 0.064), *Lactobacillus* (*r* = −0.50, FDR-adjusted *p* = 0.014), *Megasphaera* (*r* = −0.63, FDR-adjusted *p* = 0.001), and *Corynebacterium* (*r* = −0.43, FDR-adjusted *p* = 0.036) were all negatively correlated with riboflavin metabolism.

### 3.7. Relationship Between Abundances of Cecal and Pulmonary Microbiota

**Figure 15:**
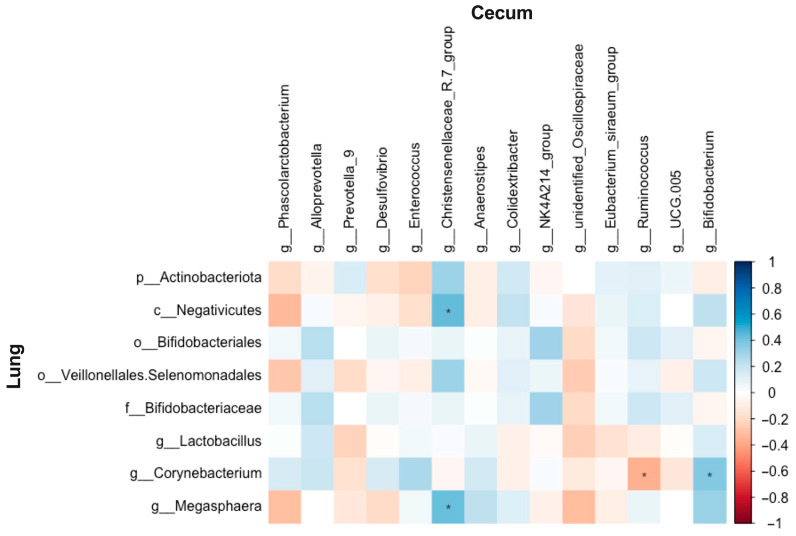
Correlations between the abundances of the altered microbial taxa in the cecum and lung of Wistar rats (*n* = 6). * means FDR-adjusted *p* < 0.1.

The abundances of the *Christensenellaceae-R7 group* showed a positive correlation with *Megasphaera* and Negativicutes (*r* = 0.43, FDR-adjusted *p* = 0.038; *r* = 0.43, FDR-adjusted *p* = 0.035, respectively). *Corynebacterium* was positively correlated with *Bifidobacterium* (*r* = 0.37, FDR-adjusted *p* = 0.072), whereas it was negatively correlated with *Ruminococcus* (*r* = −0.36, FDR-adjusted *p* = 0.086) (Figure 15).

### 3.8. Effect of BT on Cecal SCFAs

**Figure 16:**
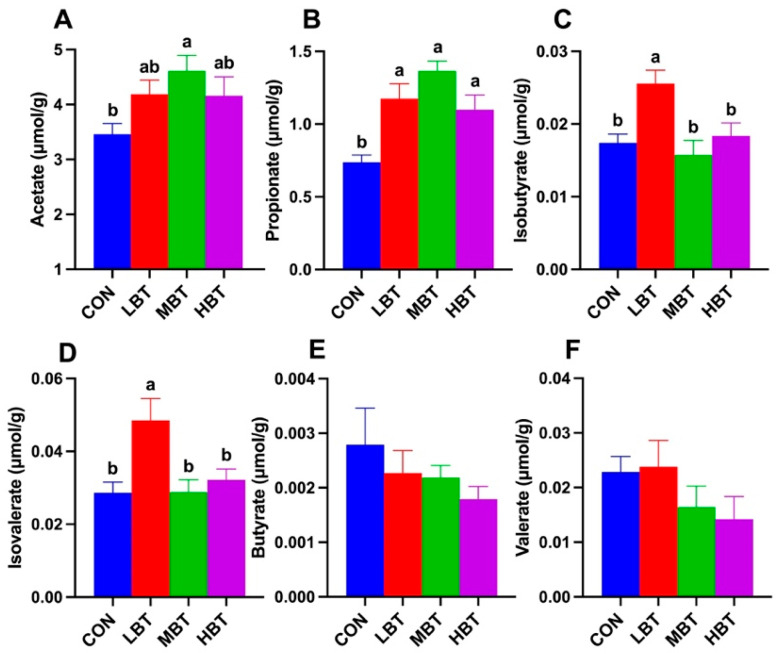
Effect of BT on contents of cecal SCFAs in Wistar rats (*n* = 10). Acetate (**A**); propionate (**B**); isobutyrate (**C**); isovalerate (**D**); butyrate (**E**); valerate (**F**). CON: control; LBT: low-dose BT; MBT: middle-dose BT; HBT: high-dose BT. Error bars indicate standard errors. Groups marked with different letters differ significantly (*p* < 0.05).

The acetate content in the middle-dose BT group was significantly higher than that in the control group (*F*_3,36_ = 3.036, *p* = 0.042; *p* = 0.026; Figure 16A). Similarly, the propionate content in the low-, middle-, and high-dose BT groups was significantly higher than that in the control group (*F*_3,36_ = 9.825, *p*< 0.001; *p* = 0.004, *p* < 0.001, and *p* = 0.022, respectively; Figure 16B). In addition, the contents of isobutyrate (*F*_3,36_ = 6.313, *p* = 0.001; *p* = 0.010, 0.002 and 0.027, respectively; Figure 16C) and isovalerate (*F*_3,36_ = 5.487, *p* = 0.003; *p* = 0.007, 0.008 and 0.034, respectively; Figure 16D) in the low-dose BT group were higher than those in control, middle-, and high-dose BT groups. In contrast, the contents of butyrate and valerate did not differ significantly among the four groups (*p >* 0.05; Figure 16E,F).

### 3.9. Expression of SCFA Receptor and Inflammasome Genes in Colon and Lung

**Figure 17:**
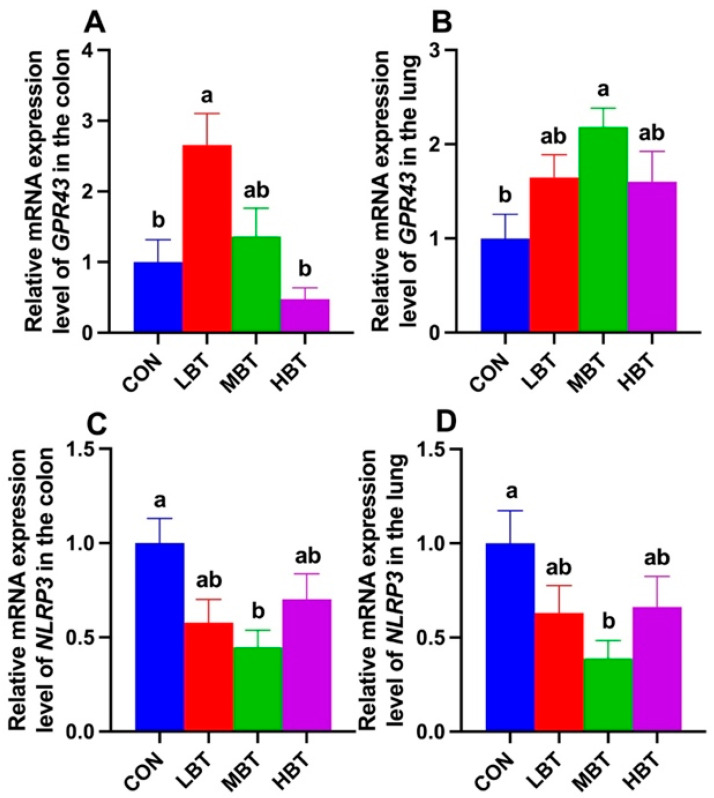
Relative mRNA expression levels of *GPR43* (**A**,**B**) and *NLRP3* (**C**,**D**) in the colon and lung of Wistar rats (*n* = 10). CON: control; LBT: low-dose BT; MBT: middle-dose BT; HBT: high-dose BT. Error bars indicate standard errors. Groups marked with different letters differ significantly (*p* < 0.05).

Relative mRNA expression level of *GPR43* in the colon of the low-dose BT group was significantly higher than in both the control and high-dose groups (*F*_3,36_ = 7.096, *p* < 0.001; *p* = 0.010 and *p* < 0.001, respectively; Figure 17A). In the lung, the relative mRNA expression of *GPR43* in the middle-dose BT group was significantly elevated compared to that in the control group (*F*_3,36_ = 3.498, *p* = 0.025; *p* = 0.013; Figure 17B). Moreover, the relative mRNA levels of *NLRP3* in the middle-dose BT group were significantly lower than those in the control group in both the colon (*F*_3,36_ = 3.835, *p* = 0.018; *p* = 0.013; Figure 17C) and the lung (*F*_3,36_ = 2.926, *p* = 0.047; *p* = 0.028; Figure 17D).

### 3.10. Effects of BT on Expressions of ZO-1 and Occludin

**Figure 18:**
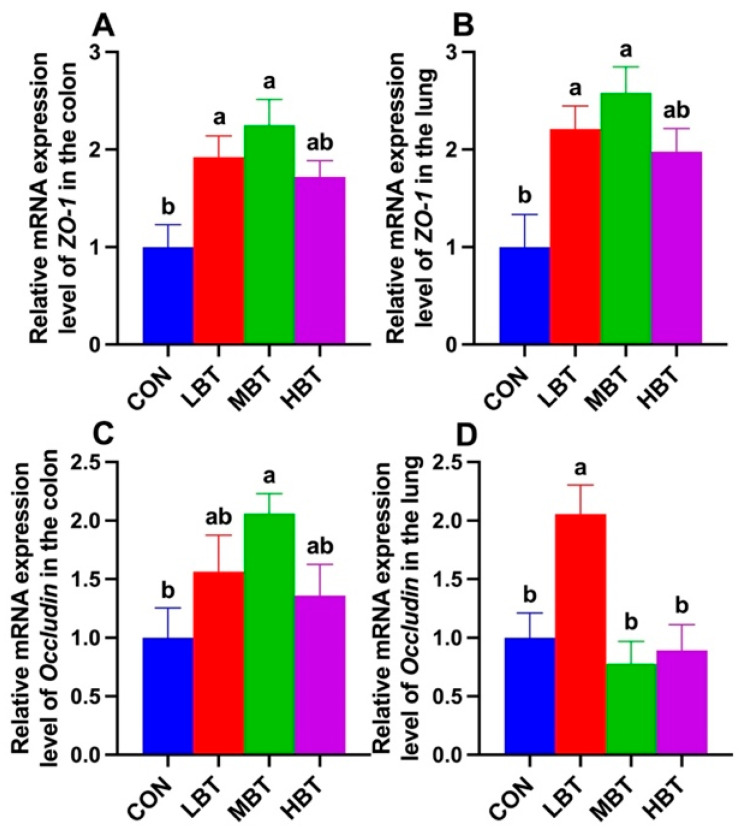
Relative mRNA expression levels of *ZO-1* (**A**,**B**) and *Occludin* (**C**,**D**) in the colon and lung of Wistar rats (*n* = 10). CON: control; LBT: low-dose BT; MBT: middle-dose BT; HBT: high-dose BT. Error bars indicate standard errors. Groups marked with different letters differ significantly (*p* < 0.05).

The mRNA expression of *ZO-1* was significantly higher in both the low-dose and middle-dose BT groups compared with the control group in the colon (*F*_3,36_ = 5.715, *p* = 0.003; *p* = 0.027 and 0.002, respectively; Figure 18A) and lung (*F*_3,36_ = 6.194, *p* = 0.002; *p* = 0.016 and 0.001, respectively; Figure 18B). Additionally, the relative mRNA expression of *Occludin* in middle-dose BT group was significantly elevated compared to the control group in the colon (*F*_3,36_ = 2.985, *p* = 0.044; *p* = 0.029; Figure 18C), whereas in the lung, the low-dose BT group exhibited a significantly higher expression level than the control group (*F*_3,36_ = 7.318, *p* < 0.001; *p* = 0.008; Figure 18D).

### 3.11. Relationship Between SCFA Content, Gene Expression, and Antioxidant and Inflammatory Levels

**Figure 19:**
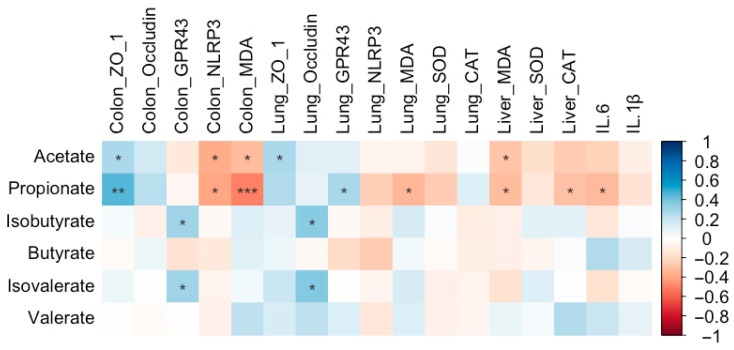
Correlations between the cecal SCFA content and antioxidant levels, expression of genes in SCFA receptor pathway, tight junction-associated gene expression, and inflammatory levels in Wistar rats (*n* = 10). * means FDR-adjusted *p* < 0.1, ** means FDR-adjusted *p* < 0.01, and *** means FDR-adjusted *p* < 0.001.

Propionate content was positively correlated with colonic *ZO-1* expression (*r* = 0.46, FDR-adjusted *p* = 0.003) and pulmonary *GPR43* expression (*r* = 0.28, FDR-adjusted *p* = 0.079) and negatively correlated with colonic *NLRP3* expression (*r* = −0.39, FDR-adjusted *p* = 0.012) and MDA level (*r* = −0.51, FDR-adjusted *p* = 0.001). Additionally, propionate content was inversely associated with MDA levels in both the liver (*r* = −0.32, FDR-adjusted *p* = 0.048) and lung (*r* = −0.34, FDR-adjusted *p* = 0.034), as well as with IL-6 levels (*r* = −0.33, FDR-adjusted *p* = 0.04) and hepatic CAT levels (*r* = −0.30, FDR-adjusted *p* = 0.61) (Figure 19). Acetate content was significantly and negatively correlated with colonic *NLRP3* expression (*r* = −0.38, FDR-adjusted *p* = 0.016), colonic MDA levels (*r* = −0.32, FDR-adjusted *p* = 0.045), and hepatic MDA levels (*r* = −0.29, FDR-adjusted *p* = 0.074) (Figure 19). In contrast, positive correlations were observed between acetate content and *ZO-1* expression in the lung (*r* = 0.28, FDR-adjusted *p* = 0.086) as well as in the colon (*r* = 0.27, FDR-adjusted *p* = 0.091). Additionally, both isobutyrate (*r* = 0.36, FDR-adjusted *p* = 0.024; *r* = 0.30, FDR-adjusted *p* = 0.057, respectively) and isovalerate (*r* = 0.38, FDR-adjusted *p* = 0.016; *r* = 0.30, FDR-adjusted *p* = 0.059, respectively) contents were positively correlated with pulmonary *Occludin* expression and colonic *GPR43* expression (Figure 19).

## 4. Discussion

This study revealed alterations in the cecal microbiota, increased production of SCFAs, and elevated expression of barrier genes (*ZO-1* and *Occludin*), accompanied by reductions in inflammatory markers (IL-1β, IL-6, and *NLRP3*) and oxidative stress following BT treatment. These findings collectively support the proposed mechanism of lung protection, which is mediated by the SCFA-driven gut–lung axis and the antioxidant properties of BT. Our current results demonstrate that oral gavage of BT for 8 weeks did not obviously change the rat weight. This might be due to the unchanged daily food intake. Similarly, the body weight gain of rats treated with burdock root ethanolic extract for 2 weeks did not vary significantly [24]. Inulin extracted from edible burdock treated for 2 weeks did not bring an obvious change in body weight in mice [25], and polyphenols from *A. lappa* treated for 4 weeks did not significantly change the body weight in mice [26]. In accordance with body weight, the weights of the lung, spleen, and liver in rats also did not change significantly. Similarly, spleen weight did not change after polyphenols from *A. lappa* treatment [26]. This indicates that BT has no obvious effect on the growth of animals. The index of liver was not reduced, which indicates that BT is not toxic to the body.

The present study found that the compounds present in BT differed from those isolated using 70% ethanol from *A. lappa* as described by Wu et al. [26], likely due to the variations in extraction methods and solvents. Nonetheless, some components, such as chlorogenic acid, caffeic acid, and ferulate, were common to both studies. L-phenylalanine, an essential amino acid, is converted into tyrosine, which then facilitates the formation of DOPA (3,4-dihydroxyphenylalanine) and dopamine, subsequently influencing blood pressure regulation [27]. Additionally, L-phenylalanine can trigger the secretion of GLP-1, thereby aiding in glucose regulation [28]. Given that L-phenylalanine was the most abundant compound detected in the present study, regular consumption of BT may help maintain stable blood glucose and blood pressure levels. Furthermore, S-Carboxymethyl-L-cysteine (CMC), a potent antioxidant used in the treatment of chronic obstructive pulmonary disease [29], was found in relatively high amounts, suggesting that BT might also support pulmonary health. In addition, D-mannose, often employed as a dietary supplement for urinary tract support, tumor growth inhibition, and regulatory T cell differentiation [30,31], further indicates that BT may confer multiple health benefits.

CAT and SOD in the antioxidant enzyme system play important roles in responding to oxidative stress [32,33]. SOD can convert superoxide radicals into hydrogen peroxide (H_2_O_2_) and molecular oxygen, while CAT transforms the harmful H_2_O_2_ into harmless water, thereby achieving antioxidant effects. MDA, a product of lipid peroxidation, reflects the extent of oxidative damage to cell membranes and is associated with the level of oxidative stress in the body [34]. In the present study, MDA levels decreased in the lung, colon, and liver, indicating that BT can reduce oxidative stress. However, CAT and SOD levels did not increase in the lung and liver; in fact, CAT levels decreased in the liver following middle- and high-dose BT treatments. We speculate that the reduction in oxidative stress may have prevented the activation of the antioxidant enzyme system, resulting in no obvious enhancement of CAT and SOD levels in the low-dose BT groups. Polyphenolic compounds, such as flavonoids, flavanols, and phenolic acids, as well as polysaccharides, can directly act as antioxidants to scavenge the free radicals to restrain the oxidative stress [35,36]. Plenty of polyphenols, flavonoids, and polysaccharides were presented in BT in the present study. CMC, quercetin, and chlorogenic acid with antioxidant properties [29,37,38] were detected in BT in the present study. Therefore, BT has the capacity as an antioxidant to directly act on free radicals to reduce oxidative stress. Similarly, polysaccharides derived from *A. lappa* could not change the CAT and SOD levels in the lung compared to the control group in mice [39], and a low dose of polyphenols from *A. lappa* did not change the serum CAT and SOD levels in mice [26]. Negative correlations between propionate and MDA levels in the lung, colon, and liver, as well as between acetate and MDA levels in the colon, indicate that SCFAs may help reduce oxidative stress. Interestingly, the observed reduction in hepatic CAT levels, along with decreases in alanine aminotransferase (ALT) and aspartate aminotransferase (AST) levels in the middle- and high-dose BT groups (unpublished data), suggests that the decline in CAT is not due to hepatotoxicity and does not imply compromised hepatic antioxidant capacity. These findings support the idea that the selected BT dosage in this study is nontoxic.

The ASV numbers identified in the lung were more than those in the cecum in the present study. This might be due to the different sampling methods and content used in the cecum, as the homogenized lung tissue method can obtain a greater microbiota signal in the lung [40]. Similarly, the number of OTUs in the lung was greater than that in the intestine of Wistar rats [41]. The two bacterial phyla with the highest abundance in the gut microbiota of rats are Firmicutes and Bacteroidetes [42]. Consistently, Firmicutes and Bacteroidetes are of the highest abundance in the cecum of rats in the present study. The Chao1 and Shannon indices were both decreased in the middle-dose group, and the microbiota community structure of both low- and middle-dose groups differed from the control group, indicating that BT gavage could influence the diversity of the cecal microbiota community with a dose-dependent effect. Consistently, polyphenols and polysaccharides from *A. lappa* treatment produced a decreasing trend in gut microbiota community diversity in mice [26,43]. The pulmonary microbiota plays a crucial role in the immune responses of both humans and animals [44]. The two most abundant bacterial phyla in the pulmonary microbiota of rats are Proteobacteria and Firmicutes [41,45]. Consistent with this, the two most abundant bacterial phyla in the pulmonary microbiota of rats in the present study were also Proteobacteria and Firmicutes. The alpha and beta diversities of the lung microbiota exhibited minimal alterations, with significant differences found in only seven taxonomic abundances—primarily within the high-dose group relative to the control group. These findings suggest that burdock root tea exerts a less pronounced effect on the diversity and structure of the lung microbiota than on the cecal microbiota community. The limited correlations observed between cecal and lung microbiota abundances confirm that these microbial communities exhibit distinct degrees of response to BT treatments. We infer that it is BT gavage directly acting on gut microbiota, while indirectly acting on lung microbiota, that brings this difference.

In the present study, administration of low- and middle-dose BT increased the relative abundances of Bifidobacteriales, Bifidobacteriaceae, and *Bifidobacterium* in the cecal microbiota of rats. Furthermore, treatment with low-dose BT selectively enriched the relative abundances of Negativicutes, Acidaminococcales, Acidaminococcaceae, *Phascolarctobacterium*, the *NK4A214 group*, *Desulfovibrio*, and *Alloprevotella*. Certain bacteria within the Bifidobacteriaceae family are capable of producing SCFAs in the human body, which in turn promote mucosal immunity, enhance barrier function, and exhibit antioxidant properties [46]. *Bifidobacterium* is recognized as a beneficial microorganism that contributes to host health by producing acetate [47,48,49]. The class Negativicutes has been confirmed to ferment carbohydrates into acetate [50]. The genus *Phascolarctobacterium*, belonging to *the* family Acidaminococcaceae, order Acidaminococcales, and class Negativicutes, is an important producer of acetate and propionate [51]. The relative abundance of intestinal *Phascolarctobacterium* increased with elevated concentrations of propionate, isobutyrate, butyrate, isovalerate, and valerate in feces [52]. The gut microbiota, such as *Alloprevotella*, contributes to intestinal development and the maturation of mucosal immunity by producing various SCFAs, thereby defending against pathogenic invasion [53]. *Desulfovibrio* metabolizes choline, ethanol, and furfural to produce acetate [54,55,56], attenuating hepatic steatosis in high-fat diet-fed mice by regulating hepatic lipid metabolism [57]. In the present study, after the intragastric administration of BT, the levels of acetate, propionate, isobutyrate, and isovalerate in the cecum increased, especially in low- and middle-dose groups. Therefore, the increments of SCFAs are due to the increased abundance of SCFA-producing bacteria. Consistently, polyphenols from *A. lappa* treatment increase the level of propionate [26]. Additionally, the *NK4A214 group* is considered to possess cellulose-degrading capabilities [58]. Therefore, BT can enrich beneficial gut bacteria to exert benefits in SCFA production and cellulose degradation. Research has demonstrated that the relative abundance of *Ruminococcus* in the gut increases in individuals suffering from various conditions, including Crohn’s disease, intestinal stress syndrome, and diabetes [59]. Furthermore, children with a history of Kawasaki disease have been found to exhibit an increased presence of *Ruminococcus*, which is capable of producing pro-inflammatory polysaccharides that disrupt intestinal immune function [60]. Similarly, the *Christensenellaceae-R7 group* is found in higher quantities in patients with intestinal ailments such as rectal cancer [61]. In the present study, BT administration resulted in a decreased relative abundance of both cecal *Ruminococcus* and the *Christensenellaceae-R7 group* in rats. Additionally, a reduction was observed in the activity of the Epstein–Barr virus infection and pathogenic *Escherichia coli* infection pathways within the middle-dose group. Given their positive correlation with both *Ruminococcus* and the *Christensenellaceae-R7 group*, these results imply that BT may help curb the proliferation of these bacteria, thereby reducing susceptibility to related viral and bacterial infections and alleviating the host’s adverse responses associated with the overgrowth of these microbial groups.

Neomycin, kanamycin, and gentamicin biosynthesis and streptomycin biosynthesis pathways in rat cecal microbiota were both downregulated by low-dose BT in the present study. Overexpression of multiple antibiotic biosynthesis (streptomycin, validamycin, vancomycin, and tetracycline) pathways in gut microbiota was observed with more exposure to antibiotics [62]. Antibiotic exposure has been linked to the selection and survival of gut bacteria with corresponding antibiotic biosynthesis genes [62]. While these antibiotics can help fight infections, they can also locally induce dysbiosis and alter gut microbiota diversity by favoring the survival of bacteria with antibiotic resistance genes [62]. The microbiome of infants born to obese mothers was significantly enriched in streptomycin biosynthesis [63]. Thus, we infer that some dose of BT intake could restrain the production of antibiotics and the bacteria with antibiotic resistance genes, which is beneficial to the gut microbiota community and the host body. *Phascolarctobacterium*, *Desulfovibrio*, and the *NK4A214 group* were negatively correlated with neomycin, kanamycin, and gentamicin biosynthesis and streptomycin biosynthesis pathways in the present study. Therefore, BT might enhance the abundance of those bacteria to inhibit antibiotic biosynthesis. When consuming BT, the rat gut is exposed to a variety of microorganisms, which have extensive metabolic capabilities that the rat lacks. These microorganisms can biotransform compounds in BT, which may be relevant to xenobiotic metabolism. This process can convert many classes of compounds, including flavonoids, isoflavonoids, lignans, phenolic acids, fiber, and tannins [64,65]. In the present study, the enrichment of benzoate degradation and styrene degradation, pathways within xenobiotic degradation and metabolism, was observed in the low-dose group. Consistently, benzoate compounds and styrene were detected in burdock root tea by LC–MC in the present study. Benzoate degradation, positively correlating with *Alloprevotella*, *Desulfovibrio*, and the *NK4A214 group*, indicates that those bacteria might participate in xenobiotic degradation and metabolism. The phenylalanine metabolism pathway, within amino acid metabolism, and pyruvate metabolism, within carbohydrate metabolism, was enriched in the low-dose group, indicating that BT can influence amino acid and carbohydrate metabolism of gut microbiota. Consistently, L-phenylalanine was the most abundant ingredient in BT in this study. However, a high level of phenylalanine in serum is harmful [66]; therefore, enrichment of the phenylalanine metabolism pathway might help to maintain the normal level of phenylalanine in rats. The underlying reason why the cecal microbial community in the low-dose BT group exhibits enriched butanoate metabolism despite an absence of a corresponding increase in butyrate content remains unclear.

*Megasphaera* is recognized as a constituent of the human lung microbiome [67] and plays a beneficial role for the host by producing SCFAs [68]. Moreover, its presence correlates with a diminished activation of host inflammatory pathways, potentially contributing to the reversal of airway inflammation [69]. The presence of *Corynebacterium* is associated with reduced colonization and infection risk levels of pneumococcus in both adults and children [70]. In in vitro experiments, media containing *Corynebacterium* inhibited the growth of *Streptococcus pneumoniae* [71]. Furthermore, the abundance of *Corynebacterium* was found to be negatively correlated with the abundance of Staphylococcus aureus [72]. *Lactobacillus* colonizes various organs in the human body, metabolizing carbohydrates to produce lactic acid and protecting the host from pathogen invasion [73]. *Lactobacillus* can prevent the colonization of *Streptococcus pneumoniae* at the respiratory mucosal barrier [74]. Furthermore, *Lactobacillus* can induce Th17 and T cells, thereby reducing lung infections in tuberculosis [75]. In the present study, the administration of middle- and high-dose BT increased the relative abundance of *Corynebacterium*, and high-dose BT increased the relative abundance of *Lactobacillus* and *Megasphaera* in the lungs of rats. Therefore, BT may help prevent the invasion of pathogens and inflammation in the lungs by increasing the abundance of *Corynebacterium*, *Lactobacillus*, and *Megasphaera* in the lungs. Furthermore, inulin extracted from edible burdock could increase the growth of cecal Lactobacilli in mice [25], and polysaccharides from *A. lappa* treatment could increase gut *Lactobacillus* abundance in LPS-treated mice [43,76]. Some bacteria in the Bifidobacteriaceae family within the human body are capable of producing SCFAs, which promote mucosal immunity, barrier function, and antioxidant activity [46]. Interestingly, this study found that high-dose BT administered via gavage increased the relative abundance of Bifidobacteriaceae in the lung. These results suggest that BT may enhance pulmonary health by promoting the proliferation of Bifidobacteriaceae.

The chromosome and associated protein pathway, within replication and repair, was enriched in the middle-dose group of lung microbiota, which might indicate that burdock root tea has a protective effect to prevent bacterial genes from mutation. The riboflavin metabolism pathway and the vitamin B6 metabolism pathway were both reduced in the high-dose group. D-alanine metabolism, within the metabolism of other amino acids, was enriched in the middle-dose group of lung microbiota. Primary bile acid biosynthesis, within lipid metabolism, was enriched in the high-dose group of lung microbiota. This indicates that BT may influence the metabolism of amino acids, vitamins, and lipids in lung microbiota. Lipids are closely linked to the early source of caseation and are essential in the generation of caseous necrosis in pulmonary tuberculosis [77,78]. Therefore, BT might enhance the lipid metabolism of microbiota to prevent the occurrence of pulmonary tuberculosis. Bifidobacteriales, Bifidobacteriaceae, *Lactobacillus*, *Corynebacterium*, and *Megasphaera*, positively correlating with primary bile acid biosynthesis, indicate that those bacteria might participate in lung lipid metabolism. Interestingly, glycerolipid metabolism was also enriched in the middle-dose BT group within the cecal microbial community. These findings imply that BT could play a beneficial role in the host’s lipid metabolism. High-dose BT was effective against pulmonary bacteria, whereas low- and mid-dose BT significantly influenced most endpoints related to cecal bacteria, with high-dose BT showing no significant impact on these endpoints. These findings reveal a dose-dependent effect of BT, supporting a hormetic biphasic dose–response model characterized by low-dose stimulation and high-dose inhibition, manifesting as either a U-shaped or an inverted U-shaped curve [79,80]. Furthermore, increasing the BT dosage beyond the high-dose level may attenuate its effect on pulmonary bacteria. This nonlinear dosage effect on cecal and pulmonary bacteria could be attributed either to the differential bioavailability of BT’s active compounds at varying doses—since each compound has a unique saturation threshold for transporters, receptors, and metabolizing enzymes [81,82]—or to shifts in microbial metabolic pathways between the cecal and pulmonary communities.

In the present study, low-dose BT gavage upregulated the relative expression of *GPR43*, while middle-dose BT gavage downregulated the relative expression of *NLRP3* in the colons of rats. Middle-dose BT gavage upregulated the relative expression of *GPR43* and downregulated the *NLRP3* expression in the lungs of rats. SCFAs mainly exert their signaling effects and participate in immune regulation by activating *GPR41* and *GPR43* [83]. *NLRP3*, an inflammasome, is significantly upregulated in chronic obstructive pulmonary disease (COPD) models [84]. SCFAs (acetate, propionate, and butyrate) can enhance the expression of *GPR43* while downregulating the expression of *NLRP3*, *IL-18*, and *IL-1β*, thereby strengthening the mucosal immune function of the gut and lungs in rats with chronic obstructive pulmonary disease [85]. The levels of acetate, propionate, isobutyrate, and isovalerate in the cecum increased, and propionate, isobutyrate, and isovalerate were positively correlated with *GPR43* expression in either the colon or lung in the present study. Consequently, the variations in *GPR43* and *NLRP3* expression may be mediated by the elevation of SCFAs in the cecum following BT gavage. In accordance with the downregulation of *NLRP3* expression, reductions in IL-1β and IL-6 levels in rat serum were observed. Consistently, negative correlations between propionate and the expression of *NLRP3* in the colon and IL-6 levels, as well as between acetate and *NLRP3* expression, were observed in the present study.

Both IL-1β and IL-6 are pro-inflammatory factors and serve as key indicators for assessing the inflammation level in the body [86,87]. In the present study, both middle- and high-dose BT gavage significantly reduced the level of IL-1β and IL-6. Similarly, long-term intake of burdock oligofructose can reduce the overexpression and excessive secretion of inflammatory factors (IL-1β, IL-6, TNF-α, and MCP-1) induced by dextran sulfate sodium, alleviating intestinal inflammation [88]. An alkali-soluble polysaccharide and a water-soluble polysaccharide from *A. lappa* treatment both reduced the serum IL-1β and IL-6 levels in inflammatory mice [43,76]. This indicates that BT may contain bioactive components involved in immune modulation, which can enhance the immune response and antiviral capacity in rats. Furthermore, the water-soluble polysaccharides in burdock may also influence the bidirectional communication between the gut and the immune system [69]. Metabolites from the gut microbiota, such as SCFAs, can affect the secretion of immune cells [89]. In the present study, BT components were found to include polysaccharides, and propionate content exhibited a negative correlation with IL-6 levels. Consequently, the immunomodulatory effects of BT on rats may be attributed to its active components, such as polysaccharides, which regulate the gut microbiota. Moreover, the influence of the gut microbiota on host immune function appears to be mediated by gut metabolic products, such as SCFAs. Additionally, *Phascolarctobacterium* exhibited a negative correlation with hepatic IL-1β levels [52]. So, enriched *Phascolarctobacterium* might contribute to the decrease in IL-1β levels by enhancing SCFAs in the rats in the present study.

The gastrointestinal and respiratory barriers are important components of the epithelial barriers in the human body [90]. They serve as selective barriers that regulate the transport of gases and nutrients while maintaining a healthy microbiota and preventing pathogen invasion [91,92]. The tight junctions (TJs) between epithelial cells uphold the integrity of the mucosal barrier [93]. TJs play a crucial role in maintaining the integrity and impermeability of the intestinal barrier [94], preventing the translocation of commensal and pathogenic bacteria [95,96]. In the present study, middle-dose BT administration significantly increased the expression of TJ proteins, particularly *ZO-1*, in the colon, while low-dose BT elevated *Occludin* expression in the same tissue. In the lung, both low- and middle-dose BT enhanced *ZO-1* expression, with low-dose BT also increasing *Occludin* levels. These findings suggest that BT reinforces the integrity of both gastrointestinal and respiratory barriers, thereby offering enhanced protection for the gut and lung. Enhancement of SCFAs in mouse feces can upregulate the mRNA levels of intestinal TJs (*ZO-1* and *Occludin*) and reduce the levels of inflammatory cytokines (IL-1β, IL-6, and TNF-α) [97]. Acetate was positively correlated with ZO-1 expression in both the colon and lung, while isobutyrate and isovalerate were positively correlated with *Occludin* expression in the lung. Therefore, upregulation of *ZO-1* and *Occludin* expression in both the colon and the lung in the present study should be due to the elevation of SCFAs in the gut. Consistently, a significantly positive correlation between SCFAs and *ZO-1* and *Occludin* expression was observed in the present study. Additionally, *Phascolarctobacterium* exhibited a positive correlation with the expression of the hepatic *Occludin* [52]. So, enriched *Phascolarctobacterium* might contribute to the elevation of *Occludin* expression by enhancing SCFAs in the rats in the present study. Enhancement of the lung weight/ body weight ratio, together with TNF-α and IL-1β produced in the lung, reflected the permeability of the air–blood barrier and an increase in lung histopathological scores [98]. Therefore, the reduced trend in the lung index, together with reduced IL-1β levels, might indicate that the lung barrier has been enhanced.

Collectively, the positive correlations between SCFA content and the pulmonary expression of *GPR43*, *ZO-1*, and *Occludin*, along with the negative correlations between SCFA levels and lung MDA, suggest that BT may exert its protective effects on the lung via a gut–lung axis mediated by SCFAs. However, as this remains speculative, further experiments using *GPR43/GPR41* knockout mice and direct SCFA supplementation are warranted to directly validate this mechanism. Moreover, based on our results and a rat-to-human dosage conversion ratio of 6:1 [99], we propose that an effective and safe daily dosage range for a 60 kg human is between 30 and 60 g of BT. In the present study, the FDR-adjusted *p*-values for many microbial parameter comparisons fall between 0.05 and 0.1, indicating that the interpretation of gut and lung microbial results should be approached with caution. Moreover, given the exploratory nature of this study, future research with larger sample sizes is warranted to confirm these findings. Our findings are derived exclusively from healthy rats; consequently, it remains unknown whether BT exerts any ameliorative or protective effects in dysfunctional or inflamed animals.

## 5. Conclusions

The present study demonstrates that BT is nontoxic and comprises numerous ingredients with beneficial bioactive properties that promote overall health. Notably, BT can alter the diversity and structure of the cecal microbiota, increase the abundance of beneficial bacteria, and enrich predicted microbial functions in both the cecum and lung of normal rats. Furthermore, BT can elevate the production of SCFAs in the cecum, upregulate the expression of SCFA receptors in the colon and lung, enhance colon and lung barrier function, and downregulate the expression of the inflammatory pathway gene *NLRP3*, thereby reducing inflammation in both tissues. Consequently, SCFAs may play a crucial mediating role in regulating immune levels in rats treated with BT. Additionally, BT can mitigate oxidative stress levels in normal rats. We infer that BT might exert a protective effect on the lung through the gut–lung axis mediated by SCFAs and through the downregulation of oxidative stress by compounds with antioxidant properties. The present study implies that BT, as a daily beverage, can confer certain healthy benefits for the gut and lungs in a normal body, providing a theoretical and experimental basis for the further development and application of BT in daily life.

## Figures and Tables

**Table 1:** Effect of BT on alpha diversities of cecal microbial community in Wistar rats (data are presented as the mean ± SEM; *n* = 6).

Index of Alpha Diversity	Control	Low-Dose BT	Middle-Dose BT	High-Dose BT
Observed features	309 ± 31 ^a^	390 ± 60 ^a^	208 ± 11 ^b^	281 ± 43 ^ab^
Chao1	309.83 ± 31.53 ^a^	390.89 ± 59.48 ^a^	209.54 ± 11.63 ^b^	282.06 ± 43.11 ^ab^
Shannon	5.53 ± 0.30 ^ab^	6.03 ± 0.36 ^a^	4.53 ± 0.26 ^b^	5.07 ± 0.36 ^ab^
Simpson	0.94 ± 0.01	0.96 ± 0.01	0.90 ± 0.02	0.91 ± 0.02
Pielou	0.67 ± 0.03	0.70 ± 0.03	0.59 ± 0.03	0.63 ± 0.03

Note: Groups marked with different letters differ significantly (FDR-adjusted *p* < 0.1).

## Data Availability

The sequence data are available from the NIH Sequence Read Archive with Bioproject ID PRJNA1261695.

## Supplementary Materials

The following supporting information can be downloaded at https://www.mdpi.com/article/10.3390/biology14081064/s1. Table S1 The primers sequences for quantitative real-time PCR (qPCR) analysis in present study. Table S2 Effect of BT on food intake and organ index of Wistar rats (*n* = 10). Table S3 Differences in taxonomic profiling of cecal and pulmonary microbiota at order, family and genus levels in response to BT treatment in Wistar rats (*n* = 6). Table S4 Differences in abundance of KEGG pathways of cecal microbial community in response to BT treatment in Wistar rats (*n* = 6). Table S5 Effect of BT on alpha diversities of pulmonary microbial community of Wistar rats (*n* = 6). Table S6 Differences in abundance of KEGG pathways of pulmonary microbial community in response to BT treatment in Wistar rats (*n* = 6). Figure S1 Positive (A) and negative (B) mode of total ion chromatogram in LC–MS analysis of BT components. Figure S2 Body weight changes in Wistar rats during eight weeks of BT treatment (*n* = 10). CON: Control; LBT: Low-dose BT; MBT: Middle-dose BT; HBT: High-dose BT. Error bars indicate standard errors.
