# Supplementary material for: Burdock Tea Affects Pulmonary Microbiota and Physiology Through Short-Chain Fatty Acids in Wistar Rats
Source: Biology (Basel). 2025 Aug 16;14(8):1064. doi: 10.3390/biology14081064 (PMC12383450; doi:10.3390/biology14081064)
Supplement: Supplementary file 1 [file biology-14-01064-s001.zip › biology-3730509-supplementary.pdf]

Supplementary materials

Table S1 The primers sequences for quantitative real-time PCR (qPCR) analysis in present study.

Gene name	Primer sequence（5'-3'）
ZO-1	F：TAATGCCCGAGCTCCGATG<br>R：CCATCTTTGGACCGATTGCTG
Occludin	F：GGTGGATCCCCAGGAGGCTA<br>R：CGGTCCATCTTTCTTCGGGTT
GPR43	F：TAATCTGACCCTGGCGGACT<br>R：GCGCACACGATCTTTGGTAG
NLRP3	F：CTTGAGCAACAACGACCTGG<br>R：ACATTTCACCCAACTGTAGGC
β-actin	F：GTACCGCTGGGACCCTACAC<br>R：AGCGTGGTGATGGCGTAGAA

Table S2 Effect of BT on food intake and organ index of Wistar rats (n=10).

	Control	Low-dose group	Middle-dose group	High-dose group
Daily food consumption (g)	24.30 ± 0.67	25.10 ± 0.74	24.76 ± 0.59	24.71 ± 0.54
Liver (%)	2.86 ± 0.11	3.07 ± 0.08	3.07 ± 0.11	2.78 ± 0.09
Kidney (%)	0.74 ± 0.01	0.73 ± 0.03	0.79 ± 0.02	0.77 ± 0.02
Lung (%)	0.49 ± 0.02	0.43 ± 0.01	0.45 ± 0.02	0.46 ± 0.02
Spleen (%)	0.20 ± 0.01	0.18 ± 0.01	0.19 ± 0.01	0.19 ± 0.01

Note: Data are presented as the mean ± SEM.

Table S3 Differences in taxonomic profiling of cecal and pulmonary microbiota at order, family and genus levels in response to BT treatment in Wistar rats (n = 6).

Tissue	Microbial taxa	Control	Low-dose BT	Middle-dose BT	High-dose BT
Cecum	o_Rhizobiales	0.0001 ± 0.0001[b]	0.0038 ± 0.0027[a]	0.0008 ± 0.0005[ab]	0.0005 ± 0.0002[ab]
	o_Burkholderiales	0.0028 ± 0.0010[b]	0.0178 ± 0.0098[ab]	0.0022 ± 0.0008[b]	0.0073 ± 0.0016[a]
	o_Pseudomonadales	0.0049 ± 0.0033[a]	0.0084 ± 0.0055[a]	0.0002 ± 0.0001[b]	0.0079 ± 0.0041[a]
	o_Enterobacterales	0.0007 ± 0.00019[a]	0.0038 ± 0.00258[a]	0.0002 ± 0.00005[b]	0.0016 ± 0.00051[a]
	f_Moraxellaceae	0.0047 ± 0.0033[a]	0.0076 ± 0.0048[a]	0.0002 ± 0.0001[b]	0.0046 ± 0.0018[a]
	f_Aerococcaceae	0.0034 ± 0.0009[a]	0.0089 ± 0.0051[a]	0.0007 ± 0.0001[b]	0.0034 ± 0.0010[a]
	f_Oscillospiraceae	0.0260 ± 0.0083[ab]	0.0592 ± 0.0209[a]	0.0035 ± 0.0011[c]	0.0119 ± 0.0067[bc]
	f_Alcaligenaceae	0.0009 ± 0.0004[b]	0.0170± 0.0108[a]	0.0009 ± 0.0004[b]	0.0051 ± 0.0019[a]

	f_Enterococcaceae	0.0001 ± 0.00003^{c}	0.0018 ± 0.00038^{a}	0.0012 ± 0.00025^{ab}	0.0006 ± 0.00012^{b}
	f_Butyricicoccaceae	0.0129 ± 0.0036^{a}	0.0222 ± 0.0095^{a}	0.0083 ± 0.0026^{ab}	0.0044 ± 0.0008^{b}
	g_UCG-005	0.0070 ± 0.0031^{b}	0.0303 ± 0.0095^{a}	0.0008 ± 0.0005^{b}	0.0032 ± 0.0015^{b}
	g_Enterococcus	0.0001 ± 0.00003^{c}	0.0018 ± 0.00038^{a}	0.0012 ± 0.00025^{ab}	0.0006 ± 0.00012^{b}
	g_Anaerostipes	0.0014 ± 0.0006^{b}	0.0046 ± 0.0029^{ab}	0.0087 ± 0.0024^{a}	0.0058 ± 0.0014^{a}
	g_Colidextribacter	0.0055 ± 0.0014^{a}	0.0022 ± 0.0005^{ab}	0.0010 ± 0.0005^{b}	0.0022 ± 0.0008^{ab}
	g_unidentified_Oscillospiraceae	0.00240 ± 0.00134^{ab}	0.00250 ± 0.00177^{a}	0.00005 ± 0.00003^{c}	0.00066 ± 0.00059^{bc}
	g_ [Eubacterium]_siraeum_group	0.0018 ± 0.00067^{ab}	0.0128 ± 0.00925^{a}	0.0000 ± 0.00000^{c}	0.0003 ± 0.00030^{bc}
	g_Prevotella_9	0.0026 ± 0.00207^{ab}	0.0026 ± 0.00161^{a}	0.0000 ± 0.00000^{c}	0.0001 ± 0.00011^{bc}
	o_Rhizobiales	0.0001 ± 0.0001^{b}	0.0038 ± 0.0027^{a}	0.0008 ± 0.0005^{ab}	0.0005 ± 0.0002^{ab}
Lung	o_Veillonellales-Selenomonadales	0.0006 ± 0.0004^{b}	0.0003 ± 0.0002^{b}	0.0001 ± 0.0001^{b}	0.0048 ± 0.0020^{a}

Note: Data are presented as the mean ± SEM. Groups marked with different letters differ significantly (*P* < 0.05).

Table S4 Differences in abundance of KEGG pathways of cecal microbial community in response to BT treatment in Wistar rats (*n* = 6).

KEGG pathways	Control	Low-dose BT	Middle-dose BT	High-dose BT
Benzoate_degradation	0.0011 ± 0.00003^{b}	0.0014 ± 0.00014^{a}	0.0011 ± 0.00003^{b}	0.0012 ± 0.00005^{ab}
Biosynthesis_of_enediyne_antibiotics	8.92E-07 ± 1.79E-07^{a}	2.02E-06 ± 3.83E-07^{a}	4.52E-07 ± 5.80E-08^{b}	8.51E-07 ± 2.21E-07^{ab}
Biosynthesis_of_type_II_polyketide_backbone	4.65E-08 ± 1.91E-08^{b}	1.31E-07 ± 3.20E-08^{a}	1.52E-08 ± 1.46E-09^{b}	3.74E-08 ± 1.37E-08^{ab}
Butanoate_metabolism	0.0076 ± 0.00026^{b}	0.0089 ± 0.00036^{a}	0.0071 ± 0.00012^{b}	0.0074 ± 0.00034^{b}
Epstein-Barr_virus_infection	3.13E-07 ± 7.66E-08^{a}	5.99E-07 ± 2.56E-07^{a}	1.19E-07 ± 1.35E-08^{b}	1.70E-07 ± 5.19E-08^{b}
G_protein-coupled_receptors	9.76E-12 ± 8.91E-12^{b}	3.67E-07 ± 1.96E-07^{a}	0.00E+00 ± 0.00E+00^{b}	0.00E+00 ± 0.00E+00^{b}
Glycerolipid_metabolism	0.0035 ± 0.00008^{b}	0.0034 ± 0.00005^{b}	0.0039 ± 0.00006^{a}	0.0037 ± 0.00015^{ab}

Lipid_metabolism	0.0009 ± 0.00006[a]	0.0008 ± 0.00003[b]	0.0009 ± 0.00005[ab]	0.0009 ± 0.00004[a]
Neomycin,_kanamycin_and_gentamicin_biosynthesis	0.0005 ± 0.00001[a]	0.0004 ± 0.00001[b]	0.0005 ± 0.00001[a]	0.0005 ± 0.00001[a]
Oxytocin_signaling_pathway	6.30E-07 ± 1.47E-07[a]	1.18E-06 ± 5.06E-07[a]	2.39E-07 ± 2.85E-08[b]	3.14E-07 ± 8.45E-08[b]
Pathogenic_Escherichia_coli_infection	5.86E-07 ± 1.06E-07[ab]	1.18E-06 ± 3.47E-07[a]	1.85E-07 ± 3.06E-08c	3.72E-07 ± 1.18E-07[b]c
Pertussis	4.90E-05 ± 5.89E-06[b]	2.40E-04 ± 1.06E-04[a]	4.61E-05 ± 5.66E-06[b]	1.16E-04 ± 4.12E-05[ab]
Pentose_phosphate_pathway	0.0077 ± 0.00014[a]	0.0071 ± 0.00017[b]	0.0079 ± 0.00007[a]	0.0078 ± 0.00015[a]
Phenylalanine_metabolism	0.0015 ± 0.00016[b]	0.0022 ± 0.00019[a]	0.0015 ± 0.00005[b]	0.0016 ± 0.00014[ab]
Photosynthesis_proteins	0.0028 ± 0.00004[a]	0.0025 ± 0.00007[b]	0.0027 ± 0.00008[ab]	0.0028 ± 0.00007[a]
Protein_folding_and_associated_processing	0.0033 ± 0.00015[b]	0.0037 ± 0.00011[a]	0.0032 ± 0.00005[b]	0.0032 ± 0.00011[b]
Pyruvate_metabolism	0.0134 ± 0.00021[b]	0.0143 ± 0.00024[a]	0.0135 ± 0.00015[b]	0.0134 ± 0.00023[b]
Streptomycin_biosynthesis	0.0025 ± 0.00002[a]	0.0024 ± 0.00004[b]	0.0025 ± 0.00004[ab]	0.0025 ± 0.00003[a]
Styrene_degradation	0.0002 ± 0.00002[b]	0.0004 ± 0.00004[a]	0.0003 ± 0.00002[b]	0.0002 ± 0.00002[b]
Transport	0.0061 ± 0.00035[a]	0.0050 ± 0.00023[b]	0.0061 ± 0.00018[a]	0.0062 ± 0.00031[a]

Note: Data are presented as the mean ± SEM. Groups marked with different letters differ significantly ($P < 0.05$).

Table S5 Effect of BT on alpha diversities of pulmonary microbial community of Wistar rats (n = 6).

Index of alpha diversity	Control	Low-dose group	Middle-dose group	High-dose group
Observed features	338 ± 62	410 ± 43	386 ± 62	382 ± 20
Chao1	342.52 ± 62.46	414.78 ± 43.15	388.93 ± 62.41	385.29 ± 19.62
Shannon	4.81 ± 0.91	5.76 ± 0.37	5.34 ± 0.64	5.46 ± 0.34
Simpson	0.81 ± 0.13	0.95 ± 0.01	0.89 ± 0.07	0.94 ± 0.01
Pielou	0.57 ± 0.10	0.66 ± 0.03	0.62 ± 0.06	0.64 ± 0.03

Note: Data are presented as the mean ± SEM.

Table S6 Differences in abundance of KEGG pathways of pulmonary microbial community in response to BT treatment in Wistar rats (n = 6).

KEGG pathways	Control	Low-dose BT	Middle-dose BT	High-dose BT
Chromosome_and_associated_proteins	0.0119 ± 0.00028^{b}	0.0117 ± 0.00021^{b}	0.0128 ± 0.00018^{a}	0.0121 ± 0.00030^{ab}
Riboflavin_metabolism	0.0015 ± 0.00002^{a}	0.0015 ± 0.00004^{ab}	0.0014 ± 0.00002^{b}	0.0013 ± 0.00002^{c}
Vitamin_B6_metabolism	0.0014 ± 0.00004^{a}	0.0014 ± 0.00005^{a}	0.0013 ± 0.00002^{a}	0.0013 ± 0.00001^{b}
D-Alanine_metabolism	0.0009 ± 0.00003^{b}	0.0009 ± 0.00001^{b}	0.0011 ± 0.00004^{a}	0.0010 ± 0.00005^{ab}
Viral_proteins	0.0007 ± 0.00004^{b}	0.0007 ± 0.00003^{b}	0.0008 ± 0.00002^{a}	0.0007 ± 0.00005^{ab}
Primary_bile_acid_biosynthesis	0.0001 ± 0.00002^{b}	0.0001 ± 0.00001^{b}	0.0002 ± 0.00003^{ab}	0.0002 ± 0.00003^{a}
Vasopressin-regulated_water_reabsorption	1.04E-11 ± 1.04E-11^{b}	2.24E-09 ± 1.38E-09^{ab}	3.50E-08 ± 3.46E-08^{a}	1.58E-06 ± 1.22E-06^{a}
Synaptic_vesicle_cycle	1.04E-11 ± 1.04E-11^{b}	2.24E-09 ± 1.38E-09^{ab}	3.50E-08 ± 3.46E-08^{a}	1.58E-06 ± 1.22E-06^{a}
Neuroactive_ligand-receptor_interaction	1.46E-08 ± 3.95E-09^{b}	7.41E-08 ± 2.34E-08^{a}	2.88E-08 ± 1.94E-08^{ab}	5.52E-08 ± 1.43E-08^{a}

Note: Data are presented as the mean ± SEM. Groups marked with different letters differ significantly ($P < 0.05$).

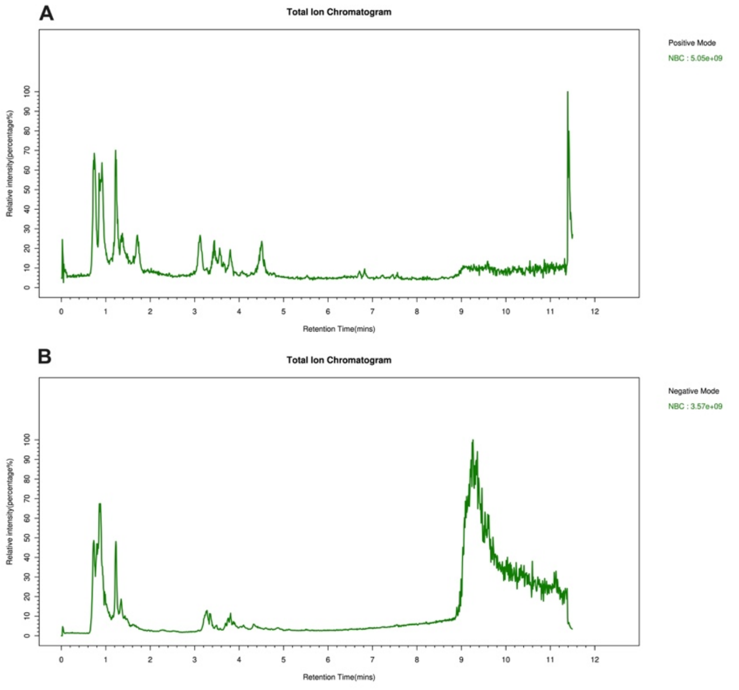

Figure S1 Positive (A) and negative (B) mode of total ion chromatogram in LC–MS analysis of BT

components.

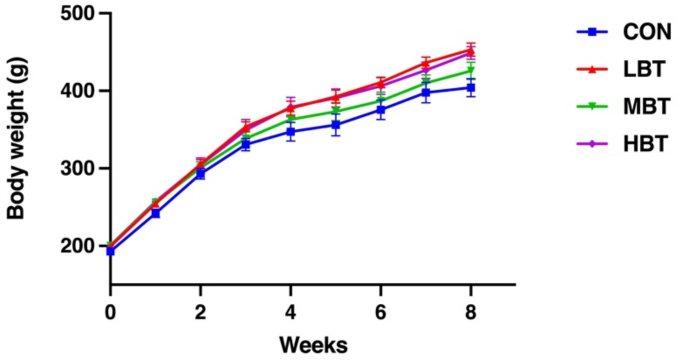

Figure S2 Body weight changes in Wistar rats during eight weeks of BT treatment (*n*=10). CON: Control; LBT: Low-dose BT; MBT: Middle-dose BT; HBT: High-dose BT. Error bars indicate standard errors.
